# Mg_3_N_2_-assisted one-pot synthesis of 1,3-disubstituted imidazo[1,5-*a*]pyridine†

**DOI:** 10.1039/c9ra10848c

**Published:** 2020-03-23

**Authors:** Suhas G. Patil, Jagannath S. Jadhav, Sagar T. Sankpal

**Affiliations:** Sant Rawool Maharaj Mahavidyalaya Kudal 416520 MS India; Department of Chemistry, Shivaji University Kolhapur 416004 MS India; Department of Chemistry, ASP College Devrukh Ratnagiri 415804 MS India dspmaspsagar@gmail.com +91 2354 260 058

## Abstract

A novel Mg_3_N_2_-assisted one-pot annulation strategy has been developed *via* cyclo-condensation reaction of 2-pyridyl ketones with alkyl glyoxylates or aldehydes, allowing the formation of imidazo[1,5-*a*]pyridines exclusively with an exellent yield.

## Graphical abstract

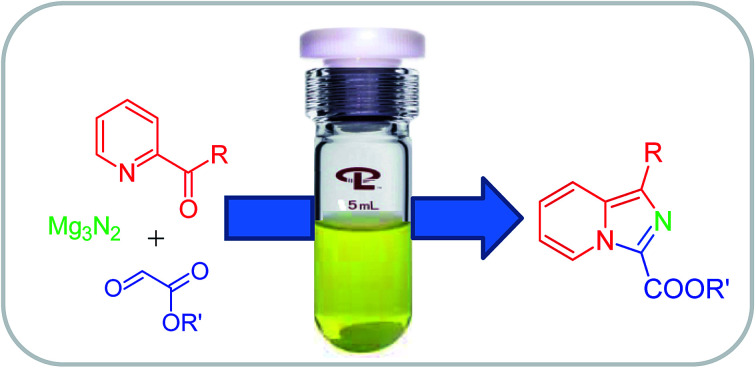

## Introduction

**Fig. 1:**
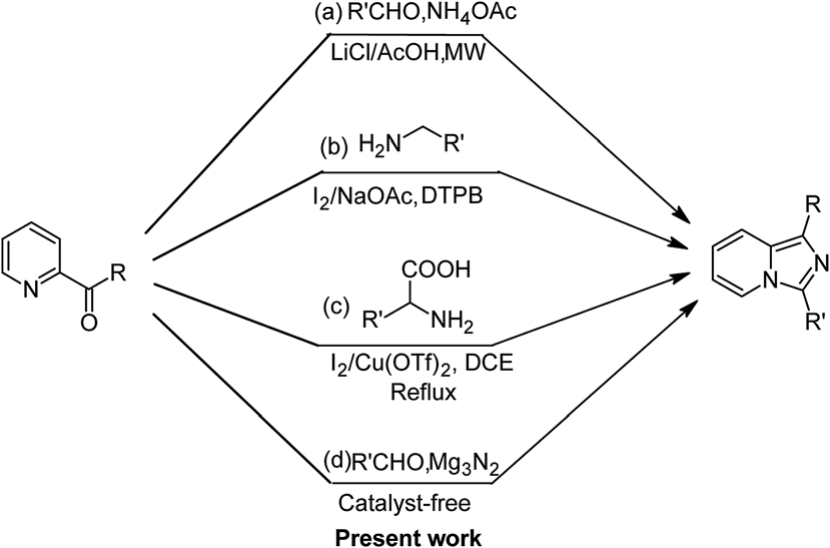
Synthetic methods for the formation of imidazo[1,5-*a*]pyridine.

The design and synthesis of new azaheterocyclic ring systems are highly necessary in modern drug discovery to achieve specific drug–receptor interactions.^1,20^ Among them, imidazo[1,5-*a*]pyridine is one of the most important and medicinally fascinating heterocyclic ring systems, which functions as a building block for the synthesis of important bio-conjugates. Additionally, it plays a crucial role in many areas of research including pharmaceuticals^2^ and materials science.^3^ Furthermore, the applications of its derivatives have also been actively probed in organic light-emitting diodes (OLED),^4^ precursors of N-heterocyclic carbenes^5^ and the design of different metal complexes.^6^ Thus, due to the superior activity of imidazo[1,5-*a*]pyridine and its derivatives, the development of new versatile and efficient protocols for their synthesis has attracted increasing interest. The existing methods^7^ mainly rely on the traditional dehydrative,^8,18^ desulfurative^9^ and oxidative^10,13*a*,19^ (Fig. 1, eqn (a)) intra-/intermolecular cyclization of 2-pyridinylmethylamine derivatives (with carbonyl compounds). Another striking tool is the direct C–H amination/cyclization strategy.^11^ The research groups of Wang^11*a*^ and Wei^11*c*^ independently investigated the synthesis of imidazo[1,5-*a*]pyridines *via* the sequential dual oxidative amination of the C sp^3^–H bonds under metal-free conditions. Moreover, the latest reports on the synthesis of imidazo[1,5-*a*]pyridine present a straightforward way to construct this particular heterocyclic ring system *via* the decarboxylative cyclic annulation of amines^12^ (Fig. 1, eqn (b)) or α-amino acids^13,19^ (Fig. 1, eqn (c)) with 2-pyridyl carbonyl compounds. Encouraged by these previous achievements and in continuation of our exploits on the development of novel approaches towards biologically active compounds,^14^ we hypothesized the development of a rapid, more practical and eco-friendly technique by utilizing a secondary nitrogen source for the shaping of the imidazo[1,5-*a*]pyridine ring structure (Fig. 1, eqn (d)).

To accomplish this goal, we searched for a substance that can act as a substitute for ammonia. A literature review revealed that magnesium nitride (Mg_3_N_2_) can act as a convenient source of ammonia when used in protic media and forms a magnesium salt with the potential to act as a catalyst.^15*a*^ Moreover, recent reports on Mg_3_N_2_ highlight its applicability in the synthesis of diverse azaheterocyclic ring systems.^15^ Thus, herein, we report a new methodology for the Mg_3_N_2_-assisted one-pot annulation reaction towards the synthesis of 1,3-disubstituted imidazo[1,5-*a*]pyridines using 2-pyridyl ketone and ethyl/methyl glyoxylates in a protic solvent. The rationale behind the use of glyoxylate is based on its high degree of electrophilic character and the production of imidazo[1,5-*a*]pyridine carboxylates that can be synthetically manipulated into complex architectures. So far, to the best of our knowledge, there is no report describing the applicability of Mg_3_N_2_ in the synthesis of imidazo[1,5-*a*]pyridinyl carboxylates.

**Scheme 1:**
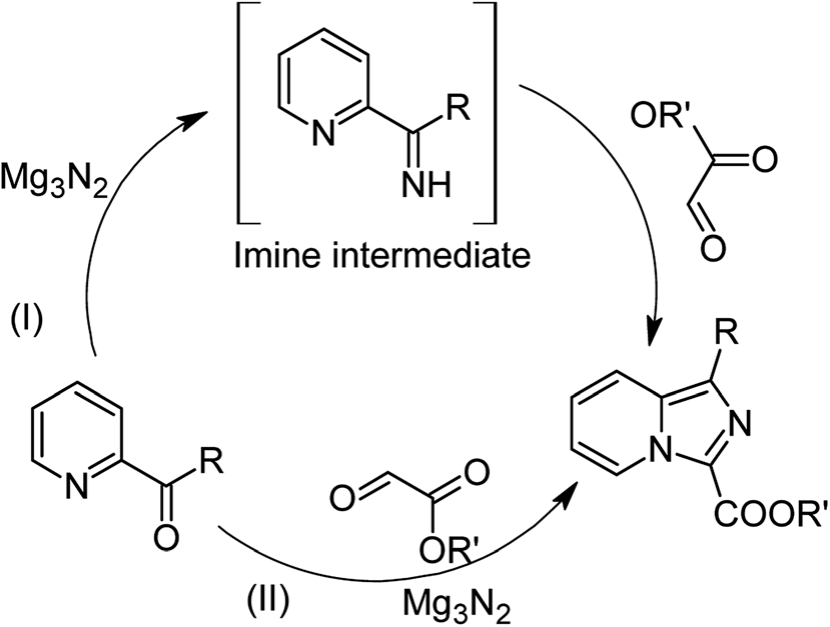
Mg_3_N_2_-assisted (I) stepwise and (II) one-pot annulation reaction for the synthesis of imidazo[1,5-*a*]pyridine.

Our study began with the two-step synthesis of commercially unavailable 2-pyridyl ketones, which were derived from the Grignard reaction of 2-pyridylmagnesium chloride and *N*,*N*-dialkyl alkyl/aryl amides according to a known procedure,^16*a*^ and their characterization data were in accordance with the literature.^16^ Having oven-ready 2-pyridyl ketone as a key substrate in hand, the next investigation commenced with establishing the best reaction conditions, where dehydrative annulation occurred smoothly. Initially, in an attempt for the stepwise formation of the desired 1,3-disubstituted imidazo[1,5-*a*]pyridines *via* the intermediate 2-pyridinylmethyl imine, we chose 2-pyridyl phenyl ketone (1a) and Mg_3_N_2_ as model substrates without their isolation, which were immediately treated for cyclization with ethyl glyoxylate (2a) (Scheme 1, route I). However, the above reaction only resulted in partial conversion under ambient conditions and at high temperatures (Table 1, entries 1–4). This indicates that the incomplete formation of ketoimine may be due to the slow evolution of ammonia or ammonia simply escaping out. Another possibility is that the complexation of the free Mg-salt with 2-imido pyridine^17^ prevents its nucleophilic attack on the aldehyde.

**Table 1:** Optimization study for the one pot synthesis of 3a

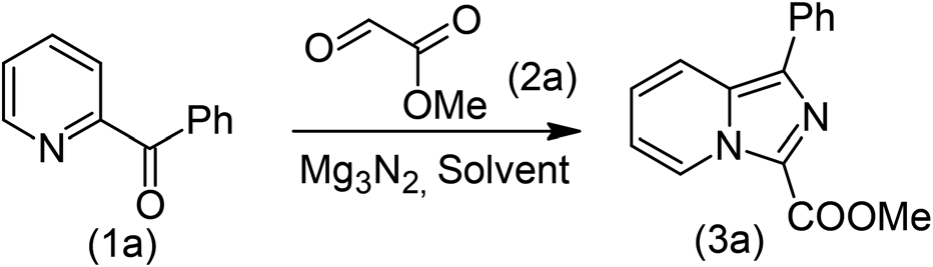				
No.	Solvent	Time (h)	Temp (°C)	% yield^a^
1	MeOH	24	25	40^b^
2	EtOH	24	25	48^b^
3	MeOH	24	60	54^b^
4	EtOH	24	75	63^b^
5	EtOH	12	25	65^c^
6	EtOH	08	60	80^c^
7	MeOH : water (8 : 2)	04	60	85^c^
8	EtOH : water (8 : 2)	04	80	92^c^
9	EtOH : water (8 : 2)	04	80	80^d^
10	EtOH : water (8 : 2)	04	80	81^e^^(^^7*c*^^)^
11	EtOH : water (8 : 2)	04	80	92^f^

a Reaction conditions: 2-pyridyl phenyl ketone (1a, 1 mol), Mg_3_N_2_ (1 mol), methyl glyoxylate (2a, 1 mol), and solvent (3 mL).

b Open flask.

c Sealed tube.

d Aq. ammonia (1 mol).

e NH_4_OAc (1 mol).

f Mg_3_N_2_ (1.5 mol).

Thus, to achieve complete conversion, in next attempt we used a sealed tube and tested the one-pot annulation (Scheme 1, route II). To our delight, the employment of a closed system ended with satisfactory conversion but extended the reaction time at ambient temperature (Table 1, entry 5). Thus, to enhance the rate of the reaction, moderate heating was applied, which stimulated the formation of 3a in excellent yields (Table 1, entry 6) and the product structure was finally unambiguously established by analyzing its IR, and ^1^H and ^13^C NMR spectra.

The results shown in Table 1 indicate that the solvent combination plays a crucial role in the present transformation (Table 1, entries 7–11). Attempts to employ other nitrogen sources (Table 1, entries 9 and 10) did not result in an improvement in percentage yield of 3a against Mg_3_N_2_, while increasing the amount of Mg_3_N_2_ to 1.5 equivalents resulted in the same yield (Table 1, entry 11). Thus, the optimal reaction conditions were achieved using 1a (1 equiv.), Mg_3_N_2_ (1 equiv.) and methyl glyoxylate 2a (1 equiv.) in EtOH : water (8 : 2) as the solvent system to obtain the maximum yield of 3a (Table 1, entry 8), and hence the same combination was chosen for further studies.

With these results in hand, we sought to examine the scope and generality of the method by employing a wide range of 2-pyridyl ketones. As shown in Table 2, this methodology tolerates a wide range of 2-pyridyl ketones (1a–k) bearing alkyl/aryl and hetero aryl functionalities. All these substrates were smoothly transformed into the expected imidazo[1,5-*a*]pyridines (3a–m) exclusively by reacting with aldehydes (2a–c) in the same pot and no anomalies were observed. Pleasingly, the sterically hindered 2-(3-methyl)pyridyl ketones (Table 2, entries 2, 4, 6, 8, and 10) also worked well under the standard conditions by affording the corresponding products in good yields. It is noteworthy that the electronic nature of the substituent on aroyl (Table 2, entries 1 and 2 and 11 and 12), heteroaroyl (Table 2, entries 3 and 4) and acyl (Table 2, entries 5–10) did not influence the rate of product formation significantly, which clearly demonstrates the high efficiency and wide generality of the present protocol. Notably, in the long run, up to five-gram-scale synthesis of 3a was achieved successfully, confirming the synthetic practicality of the present method.

**Table 2:** One-pot synthesis of imidazo[1,2-*a*]pyridine^a^

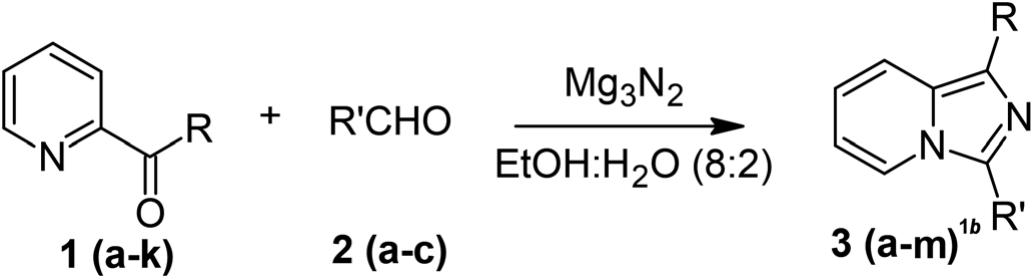 ^1*b*^					
No.	Ketone (1)	RCHO (2)	Product (3)	Reaction time (h)	% yield^b^
1	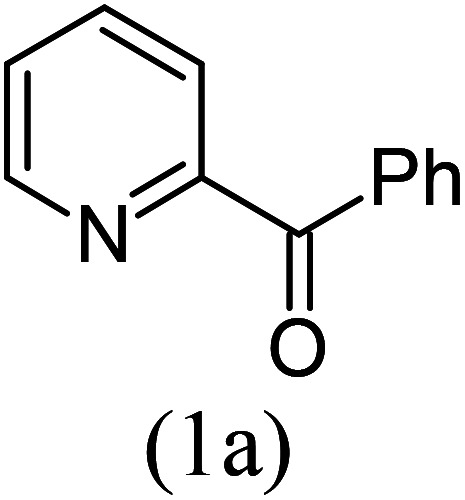	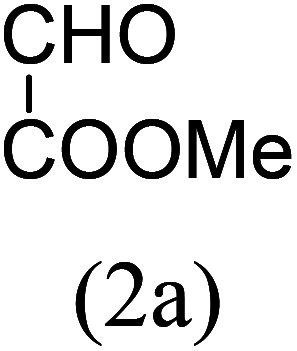	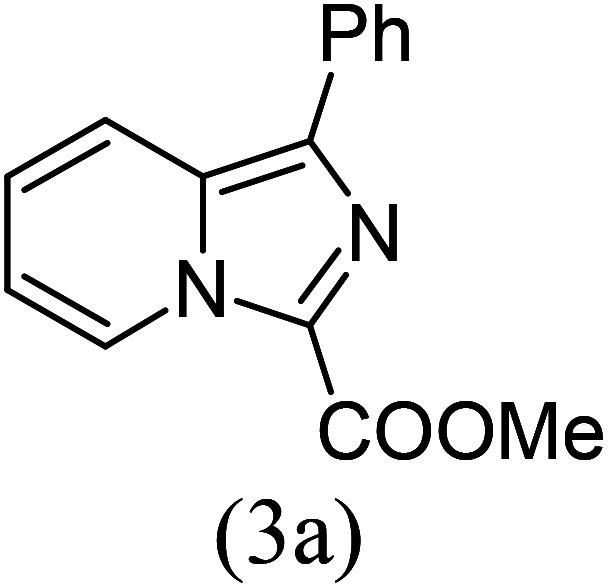	4	92
2	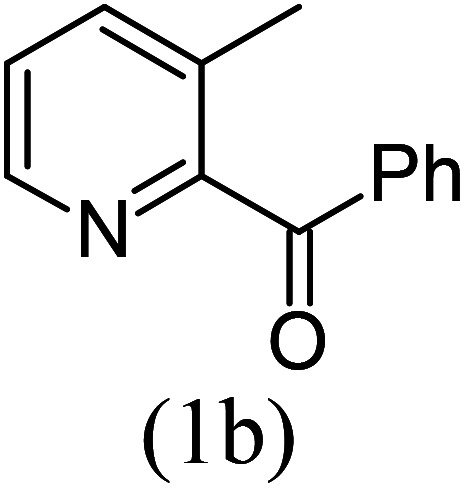	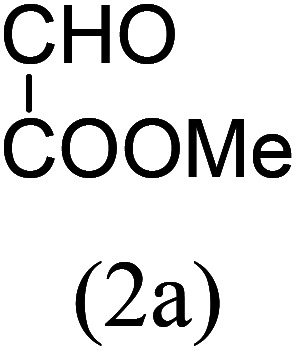	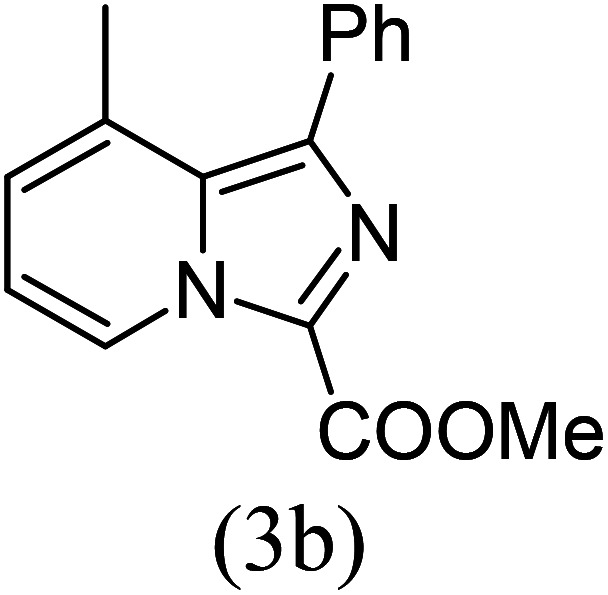	5	85
3	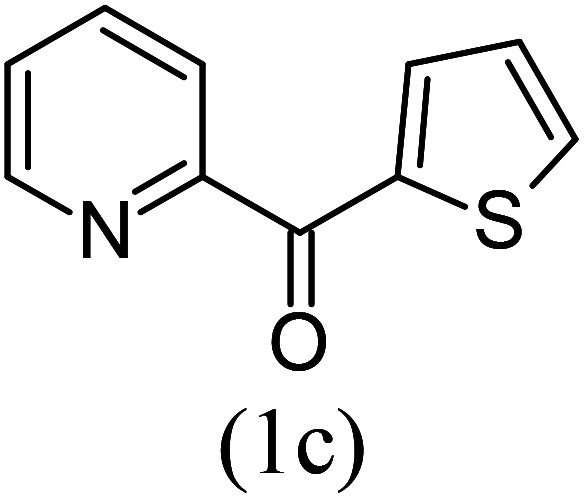	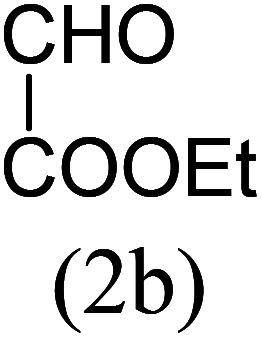	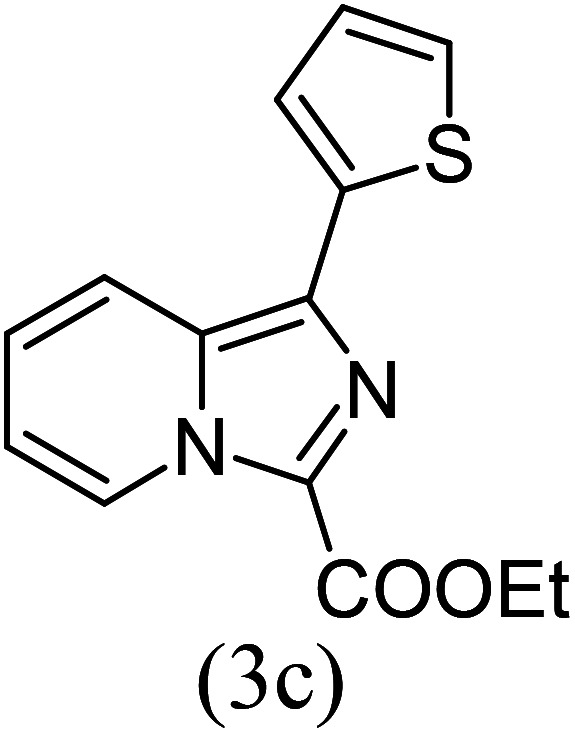	4.5	90
4	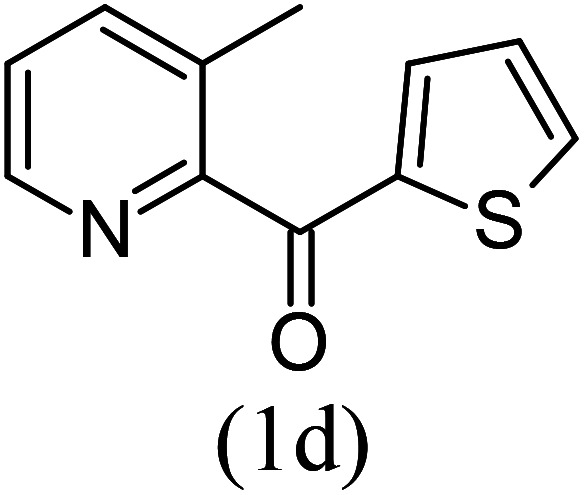	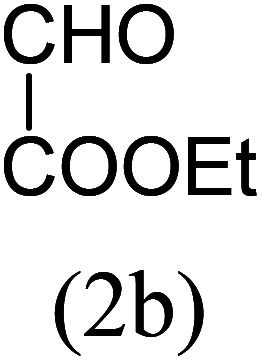	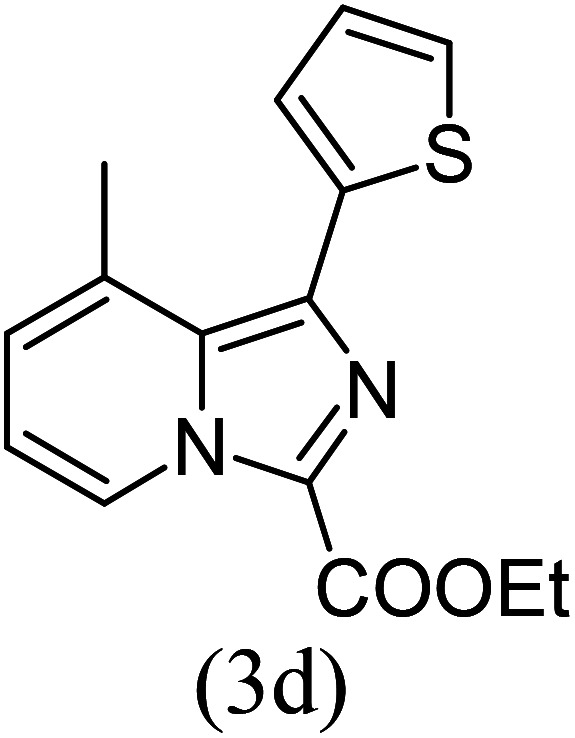	5.5	83
5	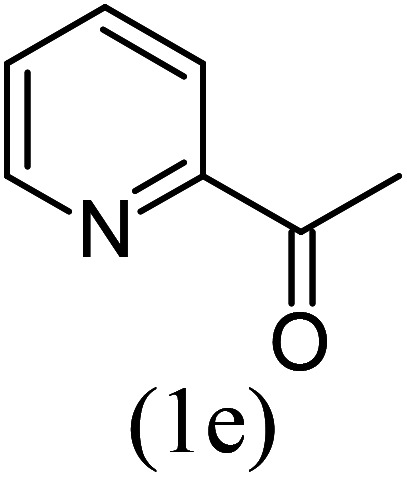	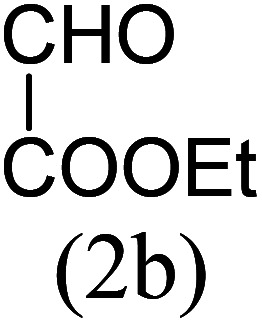	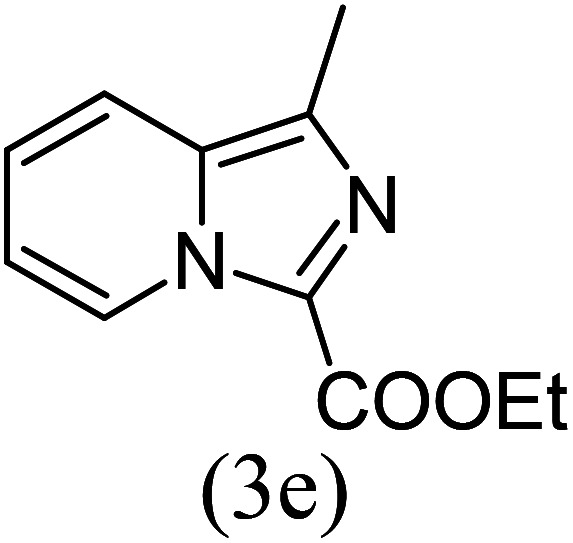	5	87
6	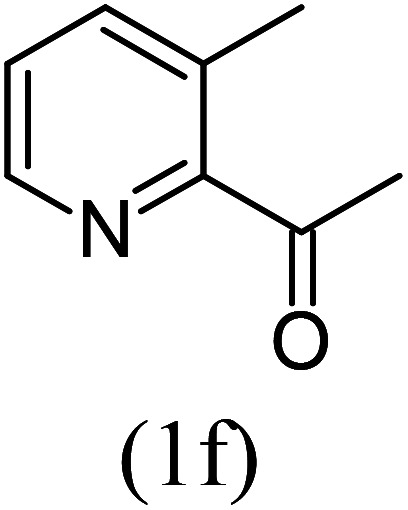	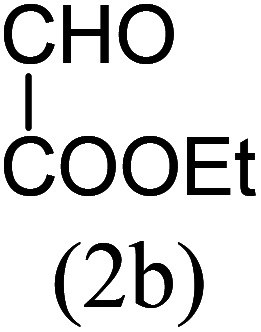	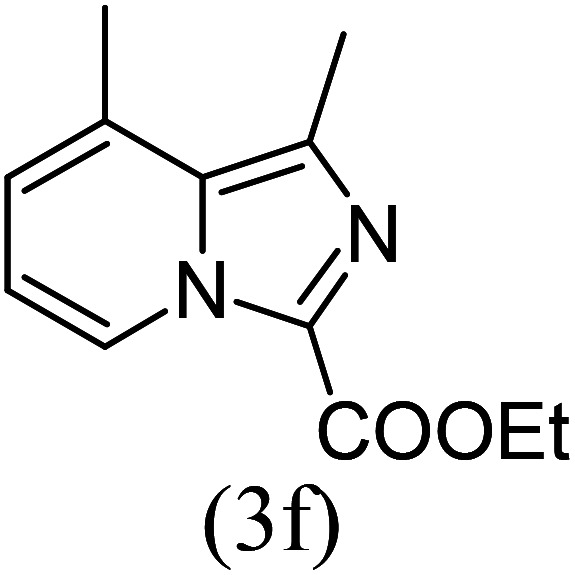	5.5	75
7	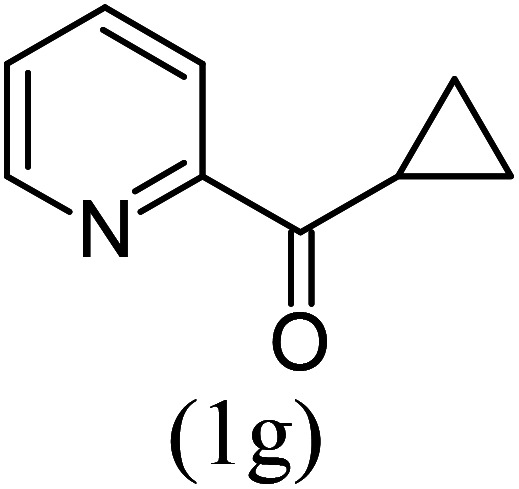	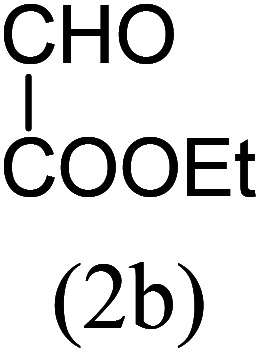	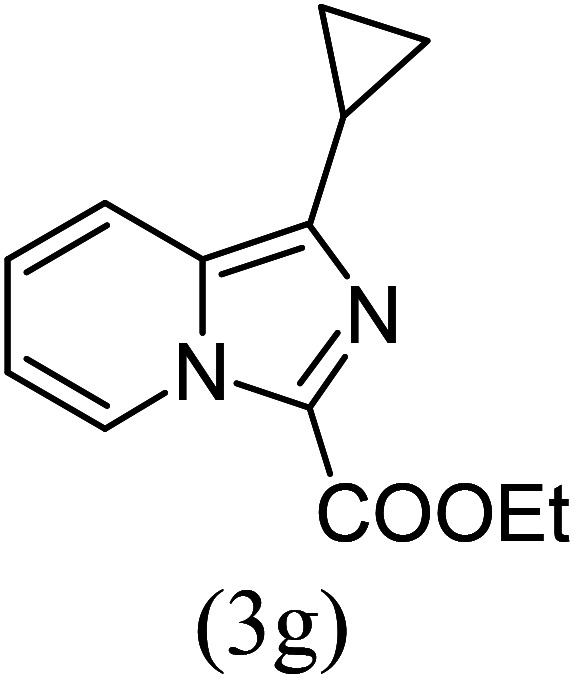	3	88
8	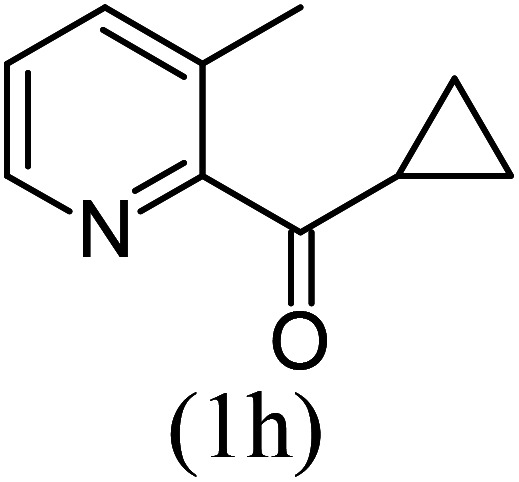	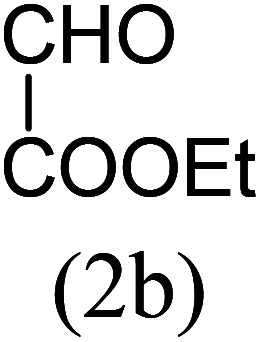	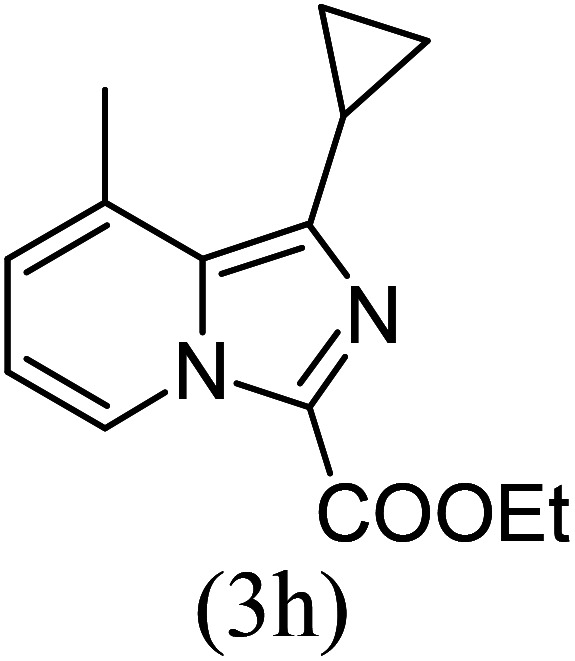	4	80
9	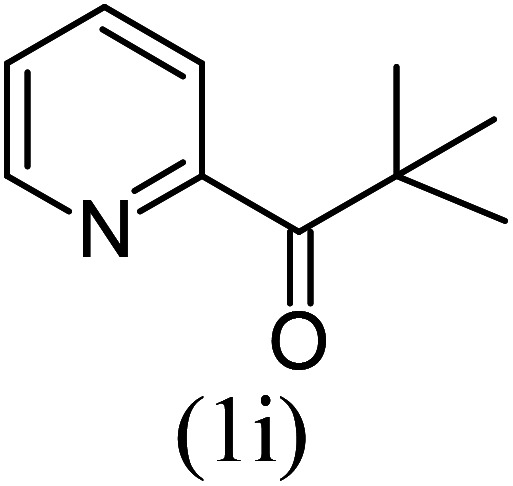	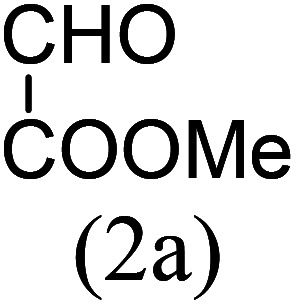	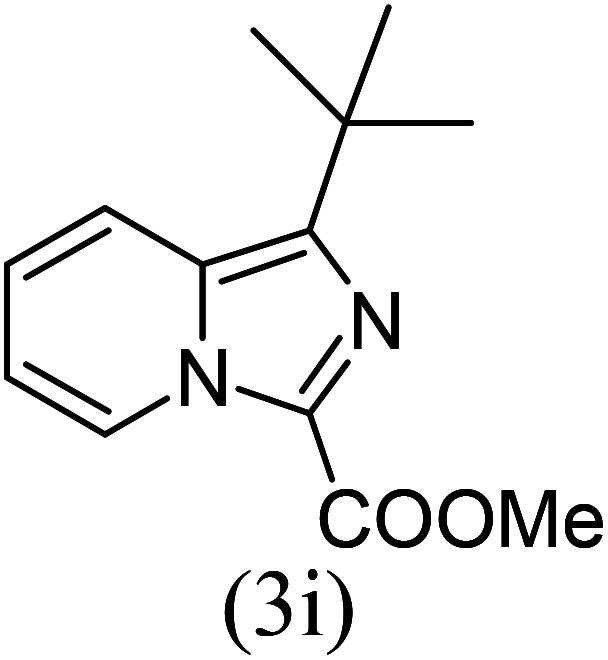	5.5	78
10	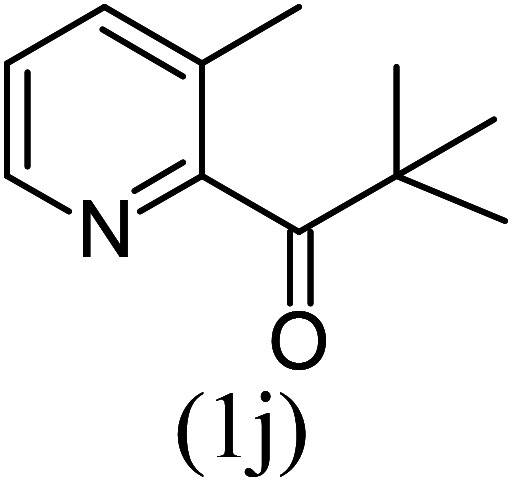	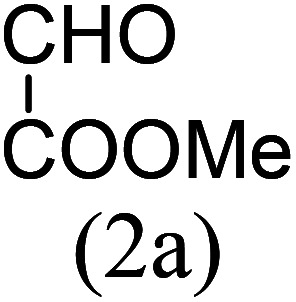	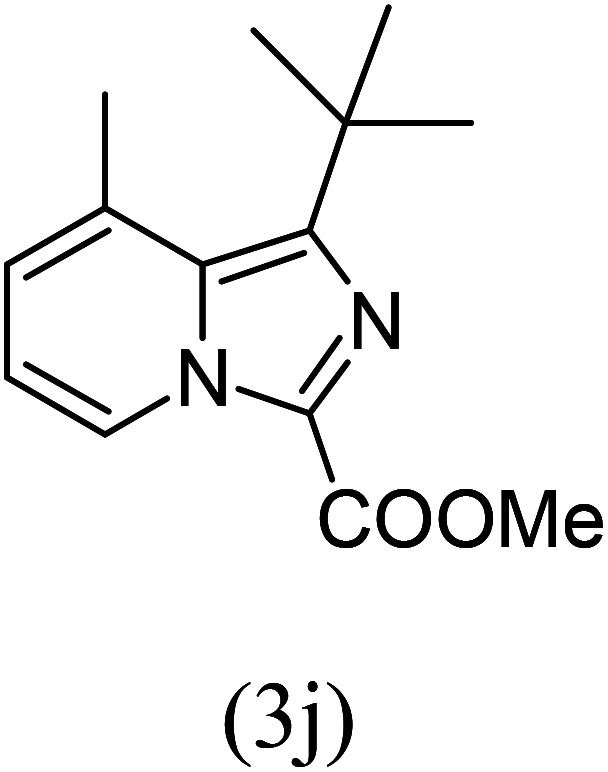	6	72
11	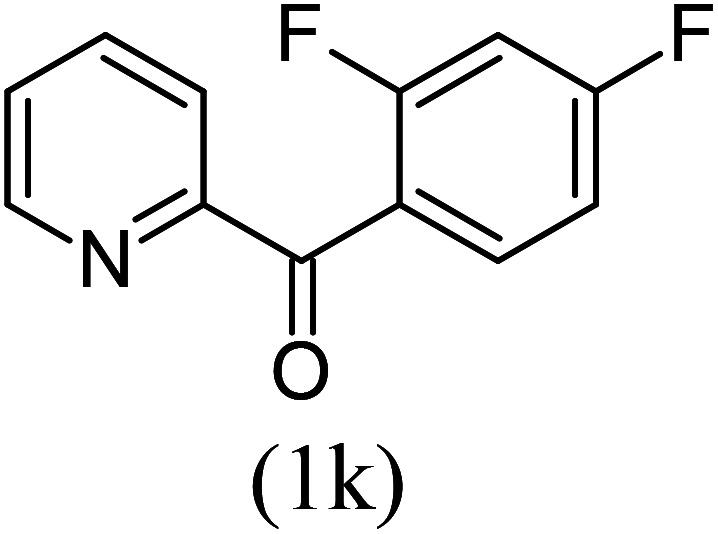	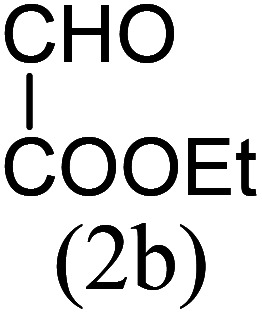	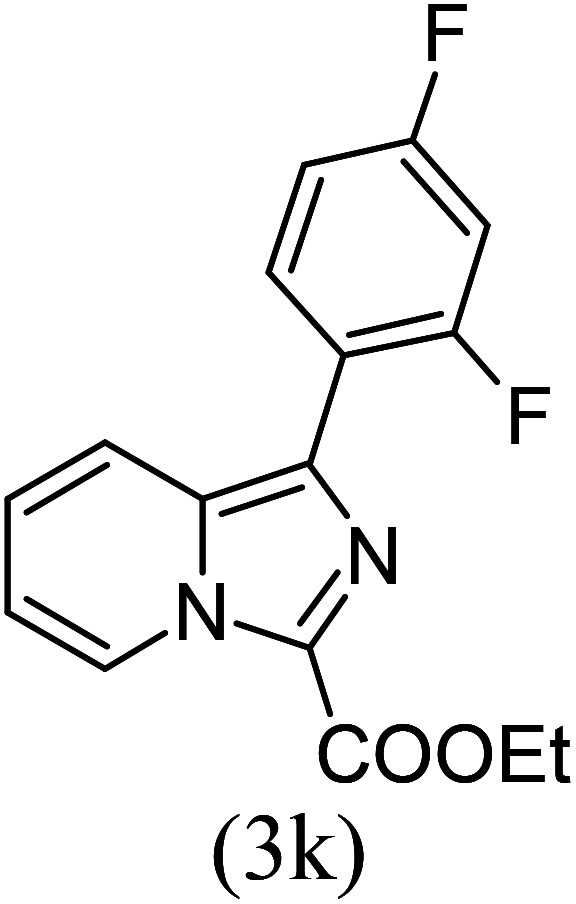	4	83
12	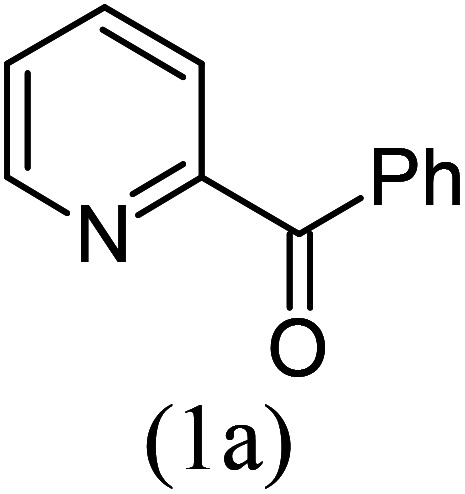	PhCHO (2c)	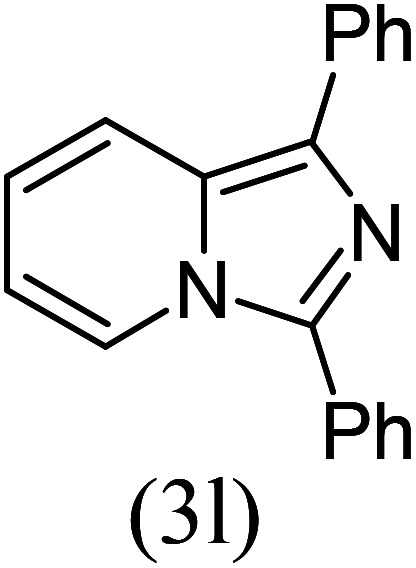	4	85 (ref. 19)
13	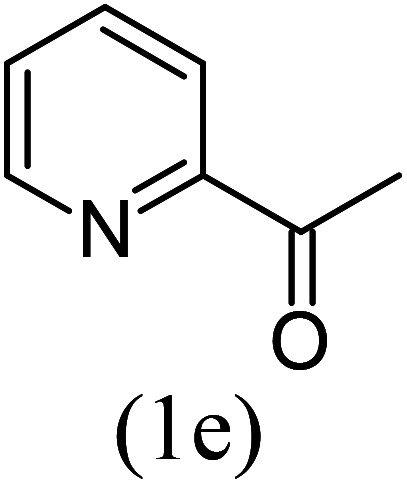	PhCHO (2c)	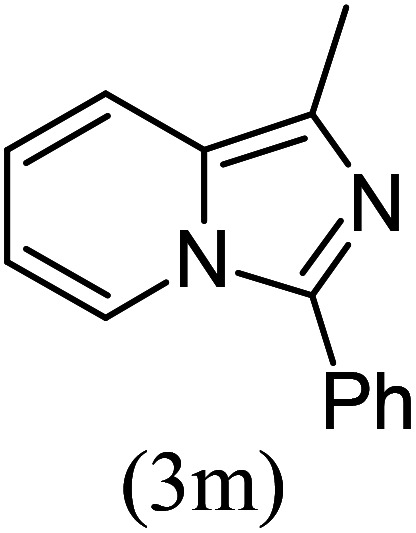	3.5	63 (ref. 18)

a Reaction conditions: 2-pyridyl ketone (1, 1 mol), Mg_3_N_2_ (1 mol), aldehyde (2, 1 mol), EtOH : water (8 : 2) (3 mL), at 80 °C.

b Isolated yields.

## Mechanism

**Scheme 2:**
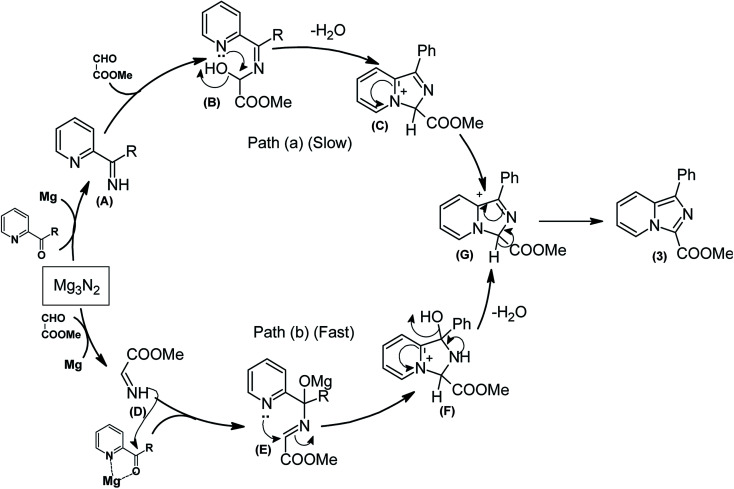
Plausible mechanistic rationalization for the synthesis of imidazo[1,5-*a*]pyridine.

Based on the abovementioned experimental results and the latest mechanistic studies on the role of Mg_3_N_2_ in azaheterocyclic chemistry,^15^ we rationalized the plausible mechanism, as depicted in Scheme 2. The reasonable explanation for the one-pot annulation between 2-pyridyl ketone and aldehyde is attributed to the simultaneous formation of two types of imines such as keto imine A (Path a) and aldimine D (Path b). Owing to the higher degree of electrophilic character of aldehyde compared to ketone, the formation of aldimines derived from ethyl glyoxylate/aldehyde seems to be very convenient, and hence Path (b) seems to be more feasible than Path (a). Consequently, the previously released Mg-salt may co-ordinates with 2-pyridyl ketone,^17*d*^ which upon graceful nucleophilic attack of aldimine D, leads to the formation of intermediate E. This upon successive intramolecular cyclization to intermediate F followed by the release of a water molecule, generates G. Finally, the intermediate G quickly tautomerizes to rearrange into the more stable imidazo[1,5-*a*]pyridine (3) as the only product.

## Conclusion

In summary, we demonstrated a novel and efficient strategy in terms of cost and ease of use for the synthesis of diverse and functionalized imidazo[1,5-*a*]pyridines through an unprecedented annulation of readily available 2-pyridyl ketones and Mg_3_N_2_ as a secondary nitrogen/amine source. We believe that any comparison between Mg_3_N_2_ and other precious amines (particularly amino acids) should indicate that Mg_3_N_2_ has valuable and unique reactivity with an entirely different set of chemical transformations, which fulfills the demands of academia and industry. Moreover, the synthesized imidazo[1,5-*a*]pyridine-3-carboxylates can be converted into more complex heterocycles *via* a systematic approach, which is ongoing in our laboratory.

## Experimental

### General information

Commercially available reagents were purchased from commercial suppliers and used without further purification. Reactions were monitored by thin layer chromatography (TLC) using MERCK precoated silica gel plates. Column chromatography was performed over silica gel (200–300 mesh). ^1^H NMR and ^13^C NMR spectra were recorded on 400 MHz NMR and 100 MHz spectrometers, respectively. Chemical shifts (*δ*) are expressed in parts per million (ppm), and coupling constants (*J*) are given in hertz (Hz). Chemical shifts are referenced to CDCl_3_ (*δ* = 7.27 for ^1^H and *δ* = 77.16 for ^13^C NMR) as an internal standard. Infrared spectra were recorded on an FTIR spectrometer. Samples were examined as KBr discs with ∼5% w/w. Elemental analyses were performed on a EURO EA3000 Vector model. Melting points were determined on a melting point apparatus and are uncorrected. High-resolution mass spectra were obtained using a GCT-TOF instrument with an ESI source.

### General procedure for the preparation of imidazo[1,5-*a*]pyridines 3(a–m)

A mixture of 2-pyridyl ketones 1 (1 mol), aldehyde 2 (1 mol) and Mg_3_N_2_ (0.150 g, 1 mol) in EtOH : water (8 : 2) (3 mL) in a 5 mL sealed tube was stirred at 80 °C for the time indicated in Table 1. Upon complete consumption of ketone 1 (monitored by TLC), the reaction mixture was allowed to cool to room temperature, quenched in ice-cold water (10 mL), and extracted with EtOAc (3 × 15 mL). The combined organic layer was dried over Na_2_SO_4_, the was solvent removed *in vacuo* and the product was purified through silica gel column chromatography using a mixture of EtOAc and *n*-hexane as the eluent to afford the corresponding product 3.

#### 1) Methyl 1-phenylimidazo[1,5-*a*]pyridine-3-carboxylate (3a)

Yellow solid; 92% yield; mp 139–141 °C; *R*_f_ 0.45 (*n*-hexane/EtOAc 8 : 2); ^1^H NMR (400 MHz, CDCl_3_) *δ* 9.42 (ddd, *J* = 6.8, 1.2, 0.8 Hz, 1H), 7.78–7.73 (m, 3H), 7.47–7.43 (m, 4H), 7.05 (td, *J* = 7.2, 1.2 Hz, 1H), 3.8 (s, 3H) ppm; ^13^C NMR (100 MHz, CDCl_3_) *δ* 161.5, 153.5, 147.1, 134.3, 130.0, 128.7, 128.3, 128.1, 127.7, 117.4, 114.2, 51.3 ppm; FT-IR (KBr, thin film): *ν* 3044.8, 2955.9, 1674.4, 1492.1, 1473.6, 1385.5, 1340.6, 1224.3, 1159.8, 917.5, 753.4, 743.3, 700.3 cm^−1^; anal. calc. for C_15_H_12_N_2_O_2_: % C, 71.42; % H, 4.79; % N, 11.10, observed: % C, 71.40; % H, 4.80; % N, 11.08; MS (EI): *m*/*z* = 252 (M^+^).

#### 2) Methyl 8-methyl-1-phenylimidazo[1,5-*a*]pyridine-3-carboxylate (3b)

Beige solid; 85% yield; mp 148–150 °C; *R*_f_ 0.50 (*n*-hexane/EtOAc 8 : 2); ^1^H NMR (400 MHz, CDCl_3_) *δ* 9.29 (dd, *J* = 6.8, 1.2 Hz, 1H), 7.76–7.74 (m, 2H), 7.47–7.41 (m, 3H), 7.26–7.24 (m, 1H), 6.99 (t, *J* = 6.8 Hz, 1H), 3.80 (s, 3H), 2.69 (s, 3H) ppm; ^13^C NMR (100 MHz, CDCl_3_) *δ* 161.6, 152.9, 147.2, 134.4, 130.1, 128.6, 127.4, 127.1, 126.0, 114.2, 51.2, 17.1 ppm; FT-IR (KBr, thin film): *ν* 2954.4, 2892.6, 1680.3, 1521.0, 1491.4, 1378.9, 1328.4, 1243.8, 1221.8, 1152.5, 784.7, 754.7 cm^−1^; anal. calc. for C_16_H_14_N_2_O_2_: % C, 72.16; % H, 5.30; % N, 10.52, observed: % C, 72.19; % H, 5.34; % N, 10.49; MS (EI): *m*/*z* = 266 (M^+^).

#### 3) Ethyl 1-(2-thionyl)imidazo[1,5-*a*]pyridine-3-carboxylate (3c)

Beige solid; 90% yield; mp 98–100 °C; *R*_f_ 0.46 (*n*-hexane/EtOAc 8 : 2); ^1^H NMR (400 MHz, CDCl_3_) *δ* 9.36 (dd, *J* = 7.2, 1.2 Hz, 1H), 8.04 (dd, *J* = 3.6, 1.2 Hz, 1H), 7.70–7.68 (m, 1H), 7.47 (dd, *J* = 4, 1.2 Hz, 1H), 7.42–7.38 (m, 1H), 7.15–7.13 (m, 1H), 7.00–6.96 (m, 1H), 4.55 (q, *J* = 7.2 Hz, 2H), 1.47 (t, *J* = 7.2 Hz, 3H) ppm; ^13^C NMR (100 MHz, CDCl_3_) *δ* 160.8, 146.8, 146.6, 136.6, 129.8, 128.5, 128.2, 128.1, 127.3, 117.1, 113.9, 110.8, 60.8, 14.4 ppm; FT-IR (KBr, thin film): *ν* 3034.6, 1694.8, 1496.0, 1446.0, 1338.4, 1231.6, 1228.1, 1157.9, 1086.3, 1046.0, 963.5, 852.9, 720.4 cm^−1^; anal. calc. for C_14_H_12_N_2_O_2_S: % C, 61.75; % H, 4.44; % N, 10.29, observed: % C, 61.79; % H, 4.40; % N, 10.25; MS (EI): *m*/*z* = 272 (M^+^).

#### 4) Ethyl 1-(2-thionyl)-8-methylimidazo[1,5-*a*]pyridine-3-carboxylate (3d)

Off-white powder; 83% yield; mp 99–101 °C; *R*_f_ 0.51 (*n*-hexane/EtOAc 8 : 2); ^1^H NMR (400 MHz, CDCl_3_) *δ* 9.23 (d, *J* = 6.8 Hz, 1H), 7.99 (dd, *J* = 3.6, 1.2 Hz, 1H), 7.47 (dd, 1H), 7.21 (d, *J* = 6.8 Hz, 1H), 7.15 (dd, *J* = 4, 1.2 Hz, 1H), 6.92 (t, *J* = 6.8 Hz, 1H), 4.50 (q, *J* = 7.2 Hz, 2H), 2.67 (s, 3H), 1.46 (t, *J* = 7.2 Hz, 3H) ppm; ^13^C NMR (100 MHz, CDCl_3_) *δ* 160.9, 147.1, 146.1, 136.8, 129.7, 127.9, 127.2, 127.1, 127.0, 126.1, 114.0, 60.6, 16.9, 14.3 ppm; FT-IR (KBr, thin film): *ν* 2984.2, 2969.7, 2945.1, 1683.0, 1458.3, 1442.7, 1394.0, 1328.0, 1271.1, 1154.9, 1068.4, 1001.0, 952.3, 928.6, 807.6, 746.3, 697.3 cm^−1^; anal. calc. for C_15_H_14_N_2_O_2_S: % C, 62.92; % H, 4.93; % N, 9.78, observed: % C, 62.95; % H, 5.00; % N, 9.80; MS (EI): *m*/*z* = 286 (M^+^).

#### 5) Ethyl 1-methylimidazo[1,5-*a*]pyridine-3-carboxylate (3e)

Off-white powder; 87% yield; mp 119–120 °C; *R*_f_ 0.41 (*n*-hexane/EtOAc 8 : 2); ^1^H NMR (400 MHz, CDCl_3_) *δ* 9.26 (d, *J* = 6.8 Hz, 1H), 7.57 (d, 1H), 7.34–7.30 (m, 1H), 6.93 (t, *J* = 6.8 Hz, 1H), 4.41 (q, *J* = 7.2 Hz, 2H), 2.67 (s, 3H), 1.41 (t, *J* = 7.2 Hz, 3H) ppm; ^13^C NMR (100 MHz, CDCl_3_) *δ* 161.3, 152.7, 146.8, 127.8, 127.4, 116.5, 113.5, 112.5, 60.2, 16.6, 14.4 ppm; FT-IR (KBr, thin film): *ν* 2979.3, 2951.4, 2928.9, 1692.5, 1497.3, 1459.1, 1385.1, 1267.0, 1153.6, 1060.0, 951.8, 879.2, 756.9, 704.1 cm^−1^; anal. calc. for C_11_H_12_N_2_O_2_: % C, 64.69; % H, 5.93; % N, 13.72, observed: % C, 64.70; % H, 5.90; % N, 13.70; MS (EI): *m*/*z* = 204 (M^+^).

#### 6) Ethyl 1,8-dimethylimidazo[1,5-*a*]pyridine-3-carboxylate (3f)

Off-white powder; 75% yield; mp 110–112 °C; *R*_f_ 0.41 (*n*-hexane/EtOAc 8 : 2); ^1^H NMR (400 MHz, CDCl_3_) *δ* 9.18 (d, *J* = 6.8 Hz, 1H), 7.18 (dt, *J* = 7.2, 1.2 Hz,1H), 6.89 (t, *J* = 7.2 Hz, 1H), 4.45 (q, *J* = 7.2 Hz, 2H), 2.74 (s, 3H), 2.62 (s, 3H), 1.43 (t, *J* = 7.2 Hz, 3H) ppm; ^13^C NMR (100 MHz, CDCl_3_) *δ* 161.6, 152.1, 147.0, 128.6, 126.4, 125.7, 113.5, 112.9, 60.2, 17.0, 16.7, 14.4 ppm; FT-IR (KBr, thin film): *ν* 2994.0, 2980.6, 2957.7, 2947.5, 1718.5, 1693.8, 1546.6, 1484.6, 1452.5, 1393.4, 1345.1, 1291.0, 1140.2, 1092.1, 1058.5, 1021.0, 748.3, 710.3 cm^−1^; anal. calc. for C_14_H_14_N_2_O_2_: % C, 66.04; % H, 6.47; % N, 12.84, observed: % C, 66.00; % H, 6.50; % N, 12.89, MS (EI): *m*/*z* = 218 (M^+^).

#### 7) Ethyl 1-cyclopropylimidazo[1,5-*a*]pyridine-3-carboxylate (3g)

Pale yellow solid; 88% yield; mp 98–100 °C; *R*_f_ 0.42 (*n*-hexane/EtOAc 8 : 2); ^1^H NMR (400 MHz, CDCl_3_) *δ* 9.31 (ddd, *J* = 7.2, 1.2, 0.8 Hz, 1H), 7.56 (dd, 1H), 7.36–7.32 (m, 1H), 6.94 (td, *J* = 7.2, 1.2 Hz, 1H), 4.48 (q, 2H), 2.87 (quin, 1H), 1.46 (t, *J* = 7.2 Hz, 3H), 1.22 (dt, 2H), 1.10 (dt, 2H) ppm; ^13^C NMR (100 MHz, CDCl_3_) *δ* 161.7, 158.1, 147.1, 127.9, 127.7, 116.4, 113.2, 60.2, 14.5, 9.9 ppm; FT-IR (KBr, thin film): *ν* 3082.4, 2984.3, 2955.6, 1675.2, 1536.8, 1413.4, 1341.6, 1088.3, 1055.8, 768.9 cm^−1^; anal. calc. for C_13_H_14_N_2_O_2_: % C, 67.81; % H, 6.13; % N, 12.17, observed: % C, 67.85; % H, 6.10; % N, 12.15; MS (EI): *m*/*z* = 230 (M^+^).

#### 8) Ethyl 1-cyclopropyl-8-methylimidazo[1,5-*a*]pyridine-3-carboxylate (3h)

Off-white powder; 80% yield; mp 105–108 °C; *R*_f_ 0.38 (*n*-hexane/EtOAc 8 : 2); ^1^H NMR (400 MHz, CDCl_3_) *δ* 9.15–9.13 (m, 1H), 7.11 (dd, 1H), 6.82 (t, *J* = 6.8 Hz, 1H), 4.47 (q, *J* = 7.2 Hz, 2H), 2.84 (quin, 1H), 2.54 (s, 3H), 1.46 (t, *J* = 7.2 Hz, 3H), 1.30–1.19 (m, 3H), 1.07–1.04 (m, 2H) ppm; ^13^C NMR (100 MHz, CDCl_3_) *δ* 161.9, 157.4, 147.4, 126.4, 125.6, 113.1, 60.1, 16.9, 14.5, 10.1 ppm; FT-IR (KBr, thin film): *ν* 2999.3, 2976.5, 2934.4, 1676.7, 1559.1, 1412.3, 1381.0, 1338.6, 1236.3, 1185.7, 1086.2, 901.6, 779.3, 756.4 cm^−1^; anal. calc. for C_14_H_16_N_2_O_2_: % C, 68.83; % H, 6.60; % N, 11.47, observed: % C, 68.80; % H, 6.65; % N, 11.50; MS (EI): *m*/*z* = 244 (M^+^).

#### 9) Methyl 1-*tert*-butylimidazo[1,5-*a*]pyridine-3-carboxylate (3i)

Pale yellow solid; 78% yield; mp 126–128 °C; *R*_f_ 0.32 (*n*-hexane/EtOAc 8 : 2); ^1^H NMR (400 MHz, CDCl_3_) *δ* 9.32 (ddd, *J* = 7.2, 1.2, 0.8 Hz), 7.71–7.68 (m, 1H), 7.38–7.34 (m, 1H), 6.98 (td, *J* = 6.8, 1.2 Hz, 1H), 3.99 (s, 3H), 1.53 (s, 9H) ppm; ^13^C NMR (100 MHz, CDCl_3_) *δ* 163.8, 161.5, 145.4, 128.2, 121.1, 117.2, 113.7, 112.1, 51.1, 34.6, 29.5 ppm; FT-IR (KBr, thin film): *ν* 2984.3, 2946.3, 1683.0, 1516.2, 1474.3, 1344.0, 1250.1, 1140.6, 1052.2, 1029.8, 914.0, 885.7, 750.3, 747.6, 722.1 cm^−1^; anal. calc. for C_13_H_16_N_2_O_2_: % C, 67.22; % H, 6.94; % N, 12.06, observed: % C, 67.20; % H, 7.00; % N, 12.10; MS (EI): *m*/*z* = 232 (M^+^).

#### 10) Methyl 1-*tert*-butyl-8-methylimidazo[1,5-*a*]pyridine-3-carboxylate (3j)

Beige solid; 72% yield; mp 112–114 °C; *R*_f_ 0.32 (*n*-hexane/EtOAc 8 : 2); ^1^H NMR (400 MHz, CDCl_3_) *δ* 9.16 (dd, *J* = 6.8, 1.2 Hz, 1H), 7.14–7.12 (m, 1H), 6.86 (t, *J* = 6.8 Hz, 1H), 3.98 (s, 3H), 2.63 (s, 3H), 1.53 (s, 9H) ppm; ^13^C NMR (100 MHz, CDCl_3_) *δ* 163.1, 161.7, 145.5, 127.2, 125.9, 113.6, 112.3, 52.9, 51.0, 34.7, 29.5, 26.1 ppm; FT-IR (KBr, thin film): *ν* 2952.4, 2929.3, 1759.2, 1714.9, 1651.1, 1474.4, 1392.6, 1343.9, 1237.7, 1185.7, 1098.3, 1074.6, 773.7, 747.1 cm^−1^; anal. calc. for C_14_H_18_N_2_O_2_: % C, 68.27; % H, 7.37; % N, 11.37, observed: % C, 68.30; % H, 7.40; % N, 11.40; MS (EI): *m*/*z* = 246 (M^+^).

#### 11) Ethyl 1-(2,4-difluorophenyl)imidazo[1,5-*a*]pyridine-3-carboxylate (3k)

Beige solid; 83% yield; mp 177 °C; *R*_f_ 0.32 (*n*-hexane/EtOAc 8 : 2); ^1^H NMR (400 MHz, CDCl_3_) *δ* 9.41 (d, *J* = 7.2 Hz,1H), 7.77 (d, *J* = 6.8 Hz, 1H), 7.64–7.52 (m, 1H), 7.49–7.42 (m, 1H), 7.10–7.07 (m, 1H), 7.01–9.98 (m, 1H), 6.93–6.88 (m, 1H), 4.31 (q, *J* = 7.2 Hz, 2H), 1.26 (t, *J* = 7.2 Hz, 3H) ppm; ^13^C NMR (100 MHz, CDCl_3_) *δ* 160.0, 155.4, 145.4, 124.4, 123.6, 111.1, 60.8, 14.4 ppm; FT-IR (KBr, thin film): *ν* 2978.4, 2939.3, 1685.6, 1497.2, 1393.4, 1337.1, 1217.9, 1169.2, 1135.9, 1043.0, 1008.6, 768.5, 753.4 cm^−1^; anal. calc. for C_16_H_12_F_2_N_2_O_2_: % C, 63.57; % H, 4.00; % N, 9.27, observed: % C, 63.60; % H, 4.05; % N, 9.30; MS (EI): *m*/*z* = 302 (M^+^).

#### 12) 1,3-Diphenylimidazo[1,5-*a*]pyridine^19^ (3l)

Yellow solid; 85% yield; mp 112–114 °C (lit. mp 110–111 °C); *R*_f_ 0.35 (*n*-hexane/EtOAc 8 : 2); ^1^H NMR (400 MHz, CDCl_3_) *δ* 8.25 (d, 1H), 7.89 (d, *J* = 5.5 Hz, 2H), 7.80 (s, 5H), 7.55 (d, *J* = 5.5 Hz, 2H), 7.50 (d, *J* = 5.5 Hz, 2H), 7.29 (s, 1H), 6.80 (s, 1H), 6.52 (s, 1H) ppm; ^13^C NMR (100 MHz, CDCl_3_) *δ* 139.0, 134.5, 132.0, 130.1, 129.0, 128.6, 128.3, 128.2, 127.5, 126.3, 126.1, 121.4, 120.0, 119.4, 113.2 ppm; FT-IR (KBr, thin film): *ν* 2978.4, 2939.3, 1655.6, 1497.2, 1393.4, 1337.1, 1217.9, 1169.2, 1135.9, 1043.0, 1008.6, 768.5, 753.4 cm^−1^; anal. calc. for C_19_H_14_N_2_: % C, 84.42; % H, 5.22; % N, 10.36, observed: % C, 84.49; % H, 5.20; % N, 10.40; MS (EI): *m*/*z* = 270 (M^+^).

#### 13) 1-Methyl-3-phenylimidazo[1,5-*a*]pyridine^18^ (3m)

Pale yellow oil; 63% yield; *R*_f_ 0.40 (*n*-hexane/EtOAc 8 : 2); ^1^H NMR (400 MHz, CDCl_3_) *δ* 8.23 (d, *J* = 6.8, Hz, 1H), 8.14 (s, 1H), 7.91 (d, *J* = 7.4 Hz, 2H), 7.37 (t, *J* = 7.4 Hz, 2H), 7.26 (t, *J* = 6.8 Hz, 1H), 6.97 (d, *J* = 6.8 Hz, 1H), 6.72 (t, *J* = 6.8 Hz, 1H), 2.53 (s, 3H) ppm; ^13^C NMR (100 MHz, CDCl_3_) *δ* 145.4, 143.7, 133.7, 128.8, 127.9, 126.4, 126.0, 124.6, 124.2, 112.7, 109.6, 17.1 ppm; FT-IR (KBr, thin film): *ν* 3024.2, 2982.5, 1648.0, 1495.5, 1370.1, 1256.5, 1078.9, 943.7, 741.6, 719.7, 692.2 cm^−1^; anal. calc. for C_14_H_12_N_2_: % C, 80.74; % H, 5.81; % N, 13.45, observed: % C, 80.75; % H, 5.79; % N, 13.50; MS (EI): *m*/*z* = 208 (M^+^).

## Conflicts of interest

There are no conflicts to declare.

## Supplementary Material

RA-010-C9RA10848C-s001
