# Supplementary material for: Mg3N2-assisted one-pot synthesis of 1,3-disubstituted imidazo[1,5-a]pyridine
Source: RSC Adv. 2020 Mar 23;10(20):11808–15. doi: 10.1039/c9ra10848c (PMC9050600; doi:10.1039/c9ra10848c)
Supplement: RA-010-C9RA10848C-s001 [file RA-010-C9RA10848C-s001.pdf]

Electronic Supplementary Material (ESI) for RSC Advances.
This journal is © The Royal Society of Chemistry 2020

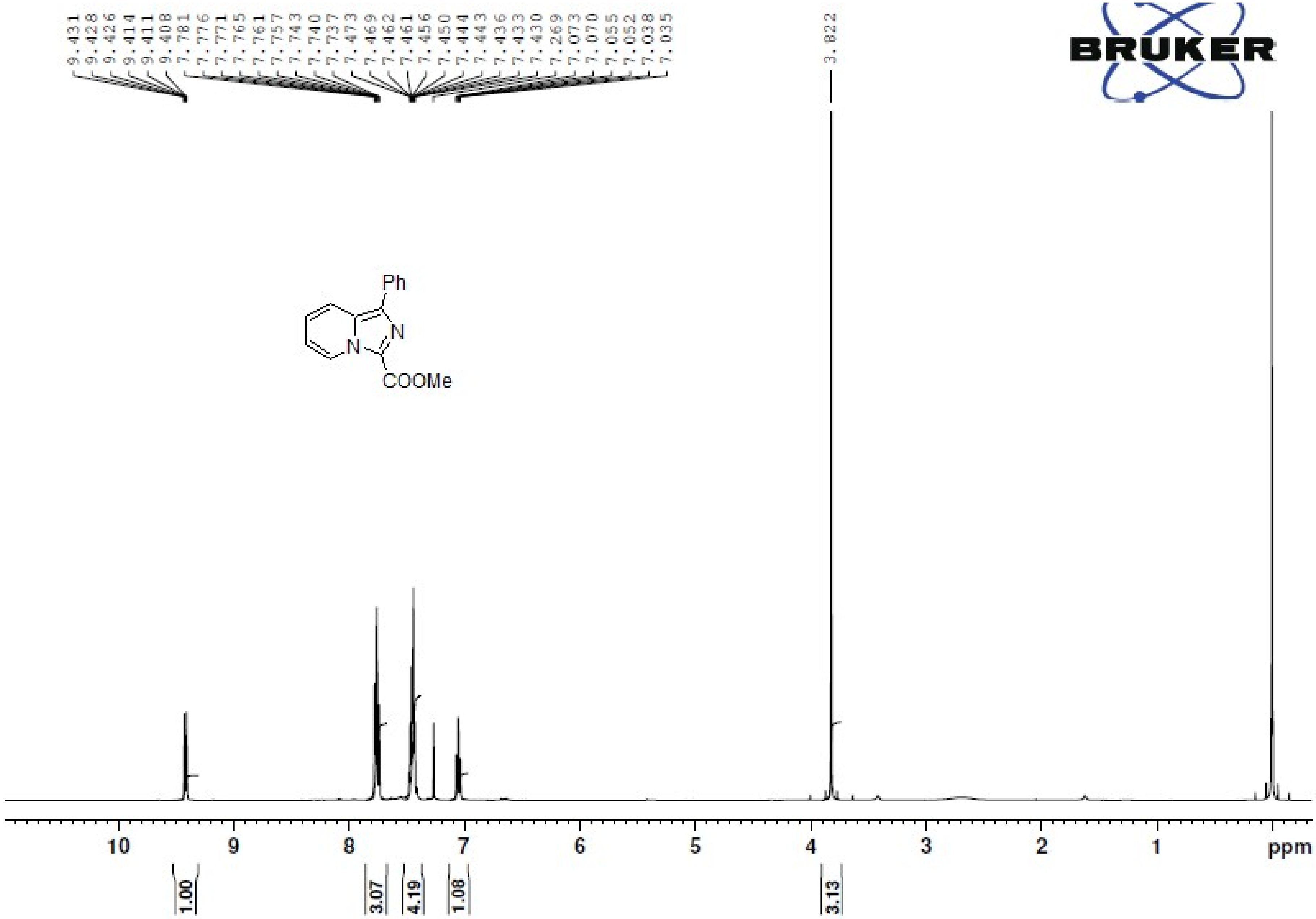

1) ^{1}H NMR spectra of Methyl 1-phenylimidazo[1,5-a]pyridine-3-carboxylate (3a)

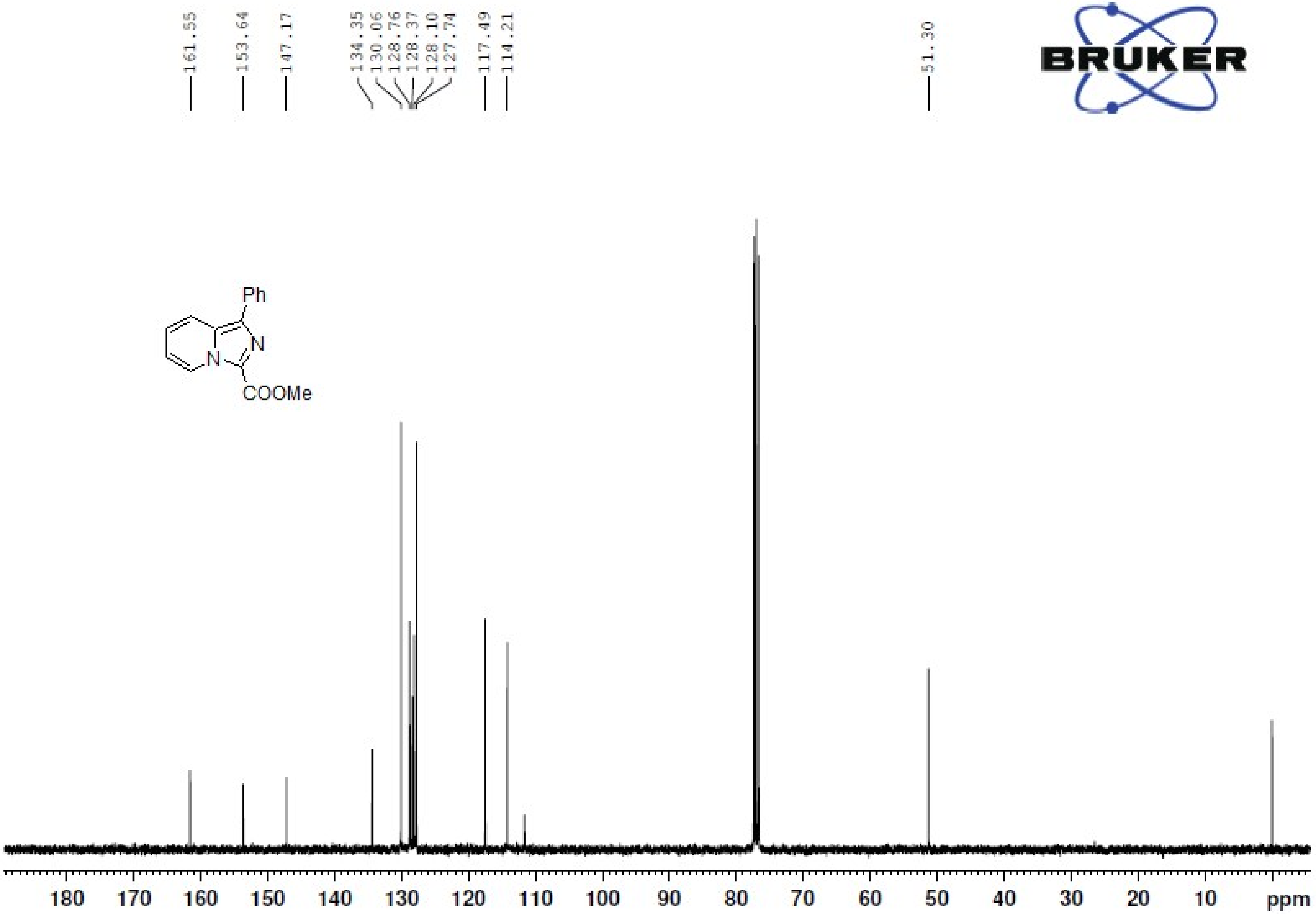

2) ^{13}C NMR spectra of Methyl 1-phenylimidazo[1,5-a]pyridine-3-carboxylate (3a)

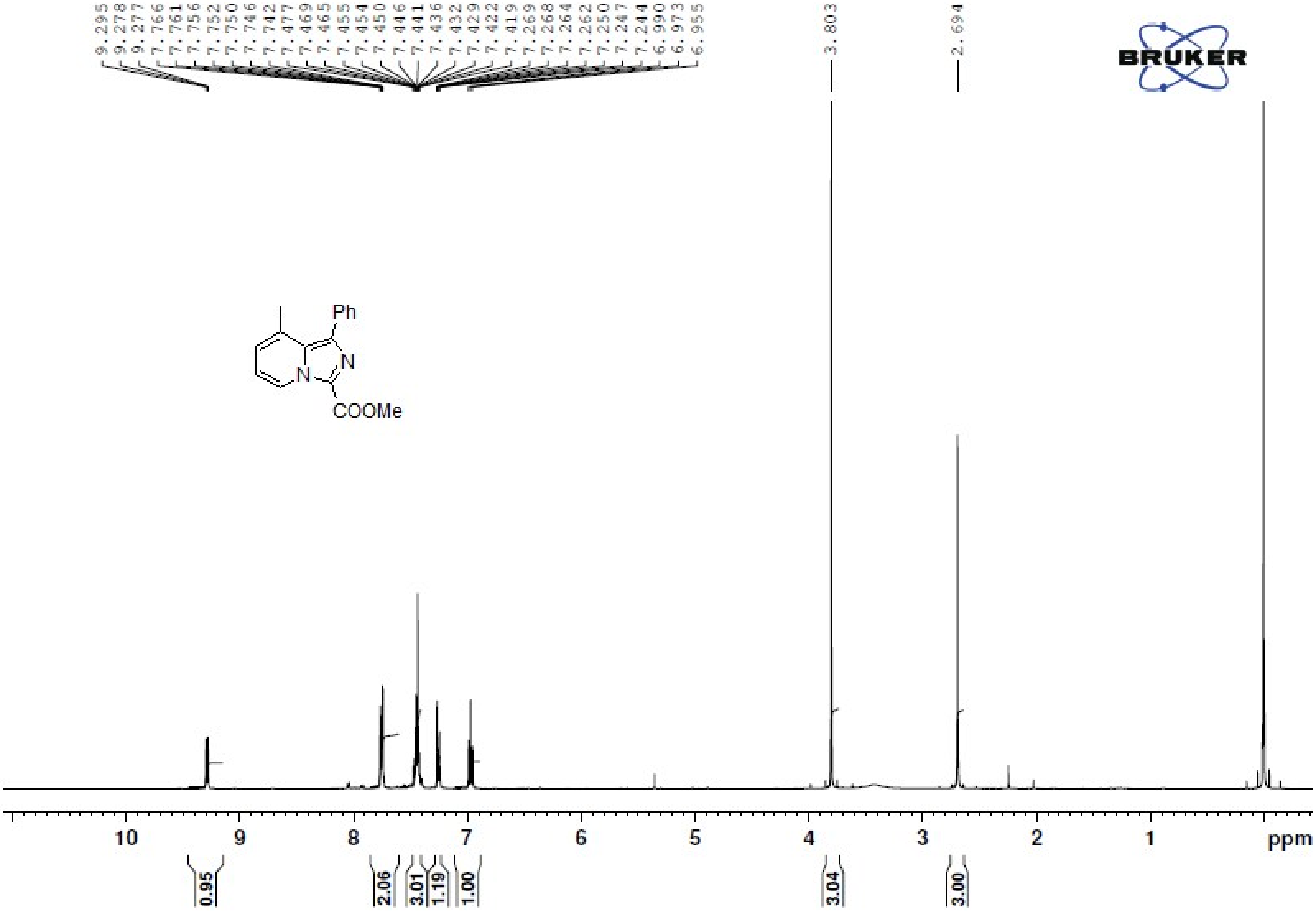

3) ^{1}H NMR spectra of Methyl 8-methyl-1-phenylimidazo[1,5-a]pyridine-3-carboxylate (3b)

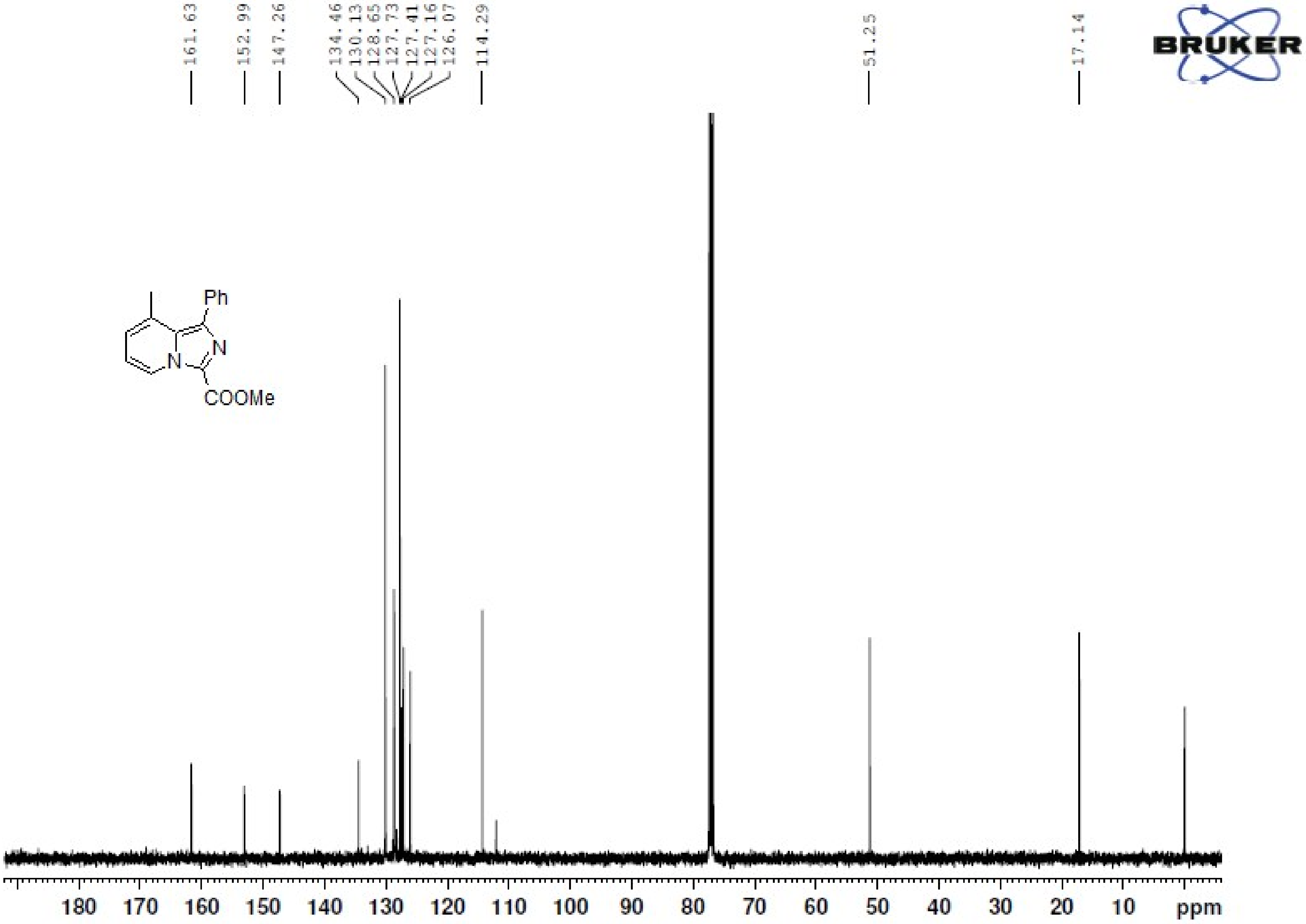

4) ^{13}C NMR spectra of Methyl 8-methyl-1-phenylimidazo[1,5-a]pyridine-3-carboxylate (3b)

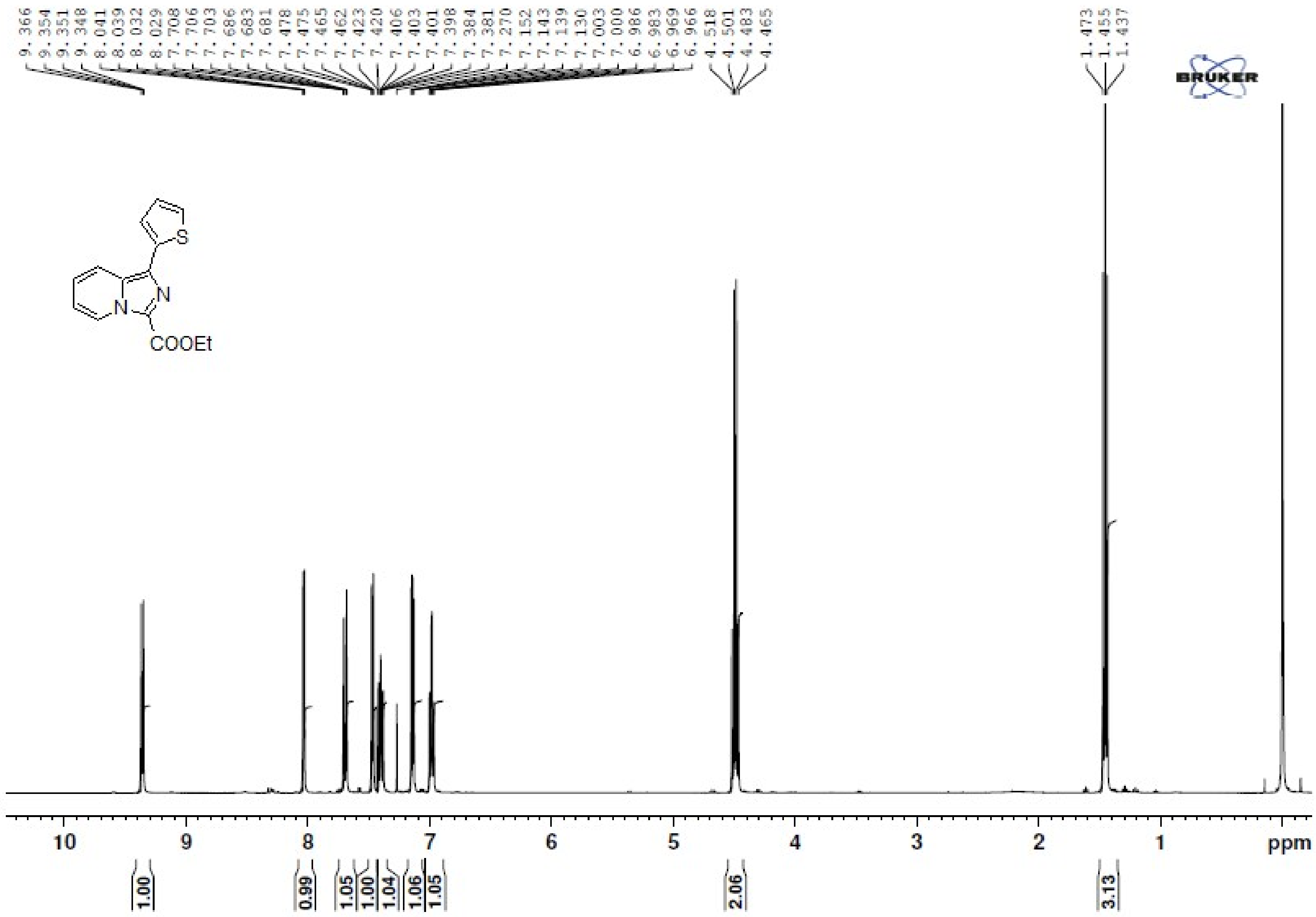

5) ^{1}H NMR spectra of Ethyl 1-(2-thionyl)imidazo[1,5-a]pyridine-3-carboxylate (3c)

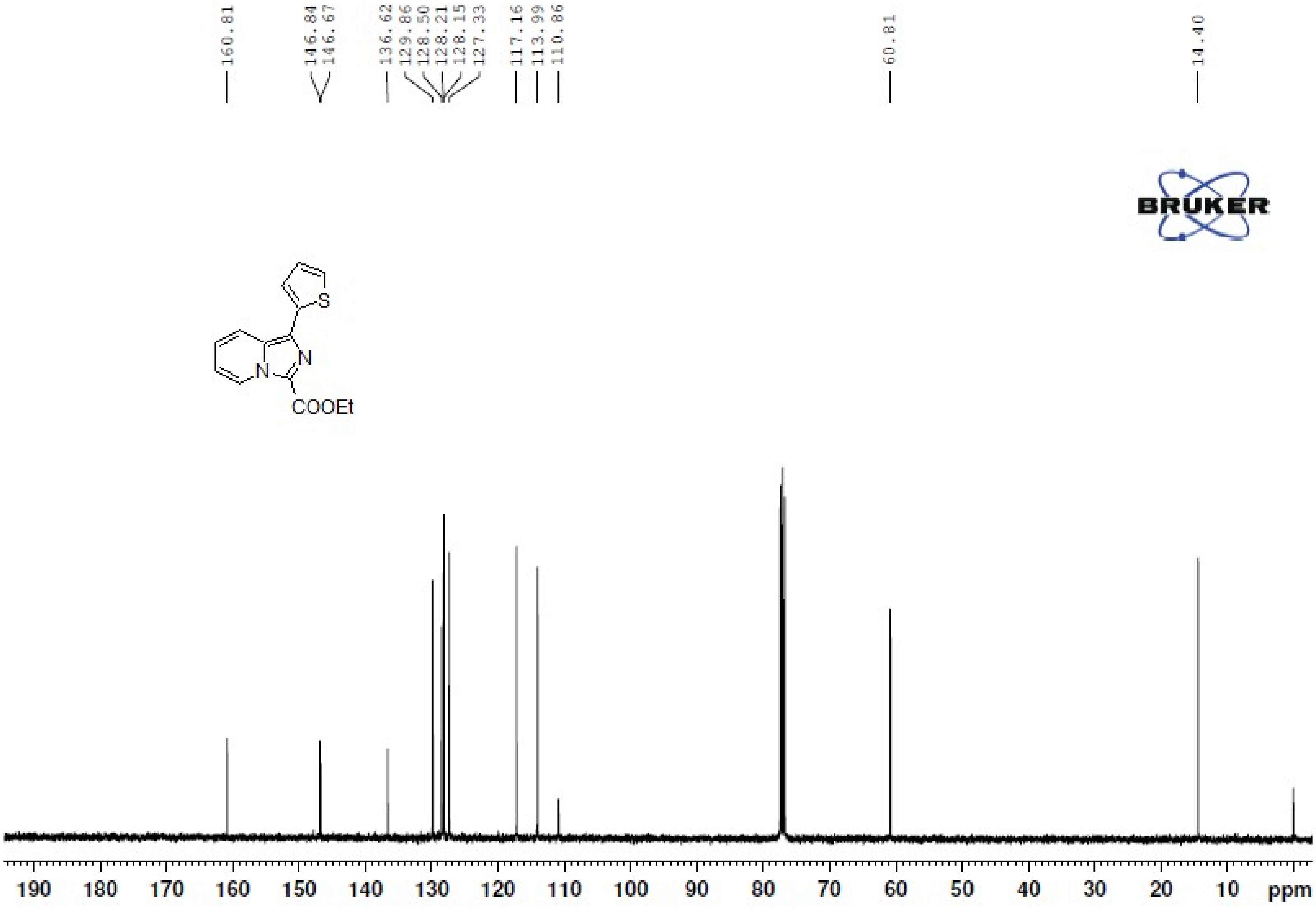

6) ^{13}C NMR spectra of Ethyl 1-(2-thionyl)imidazo[1,5-a]pyridine-3-carboxylate (3c)

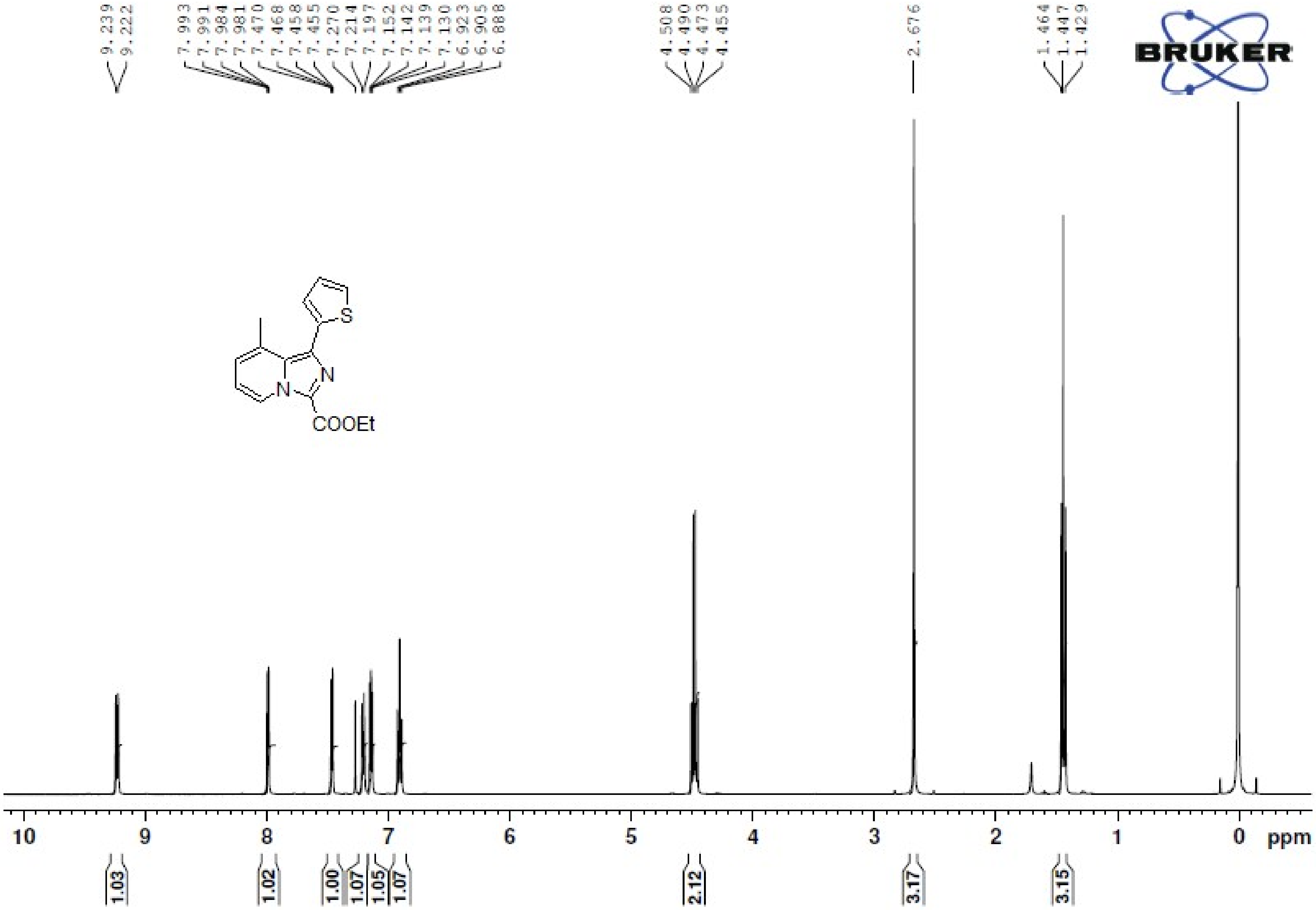

7) ^{1}H NMR spectra of Ethyl 1--(2-thionyl)-8-methylimidazo[1,5-a]pyridine-3-carboxylate (3d)

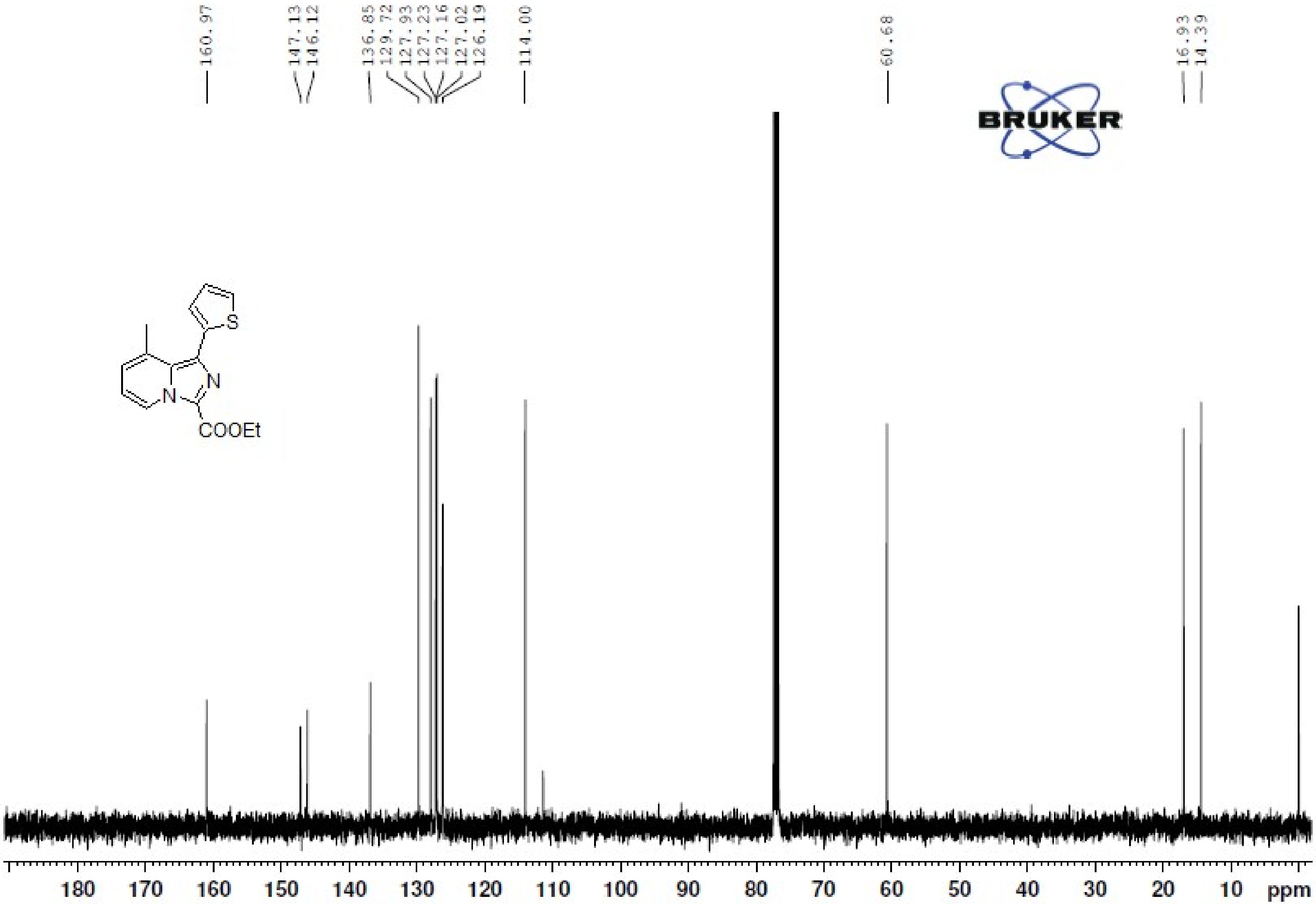

8) ^{13}C NMR spectra of Ethyl 1--(2-thionyl)-8-methylimidazo[1,5-a]pyridine-3-carboxylate (3d)

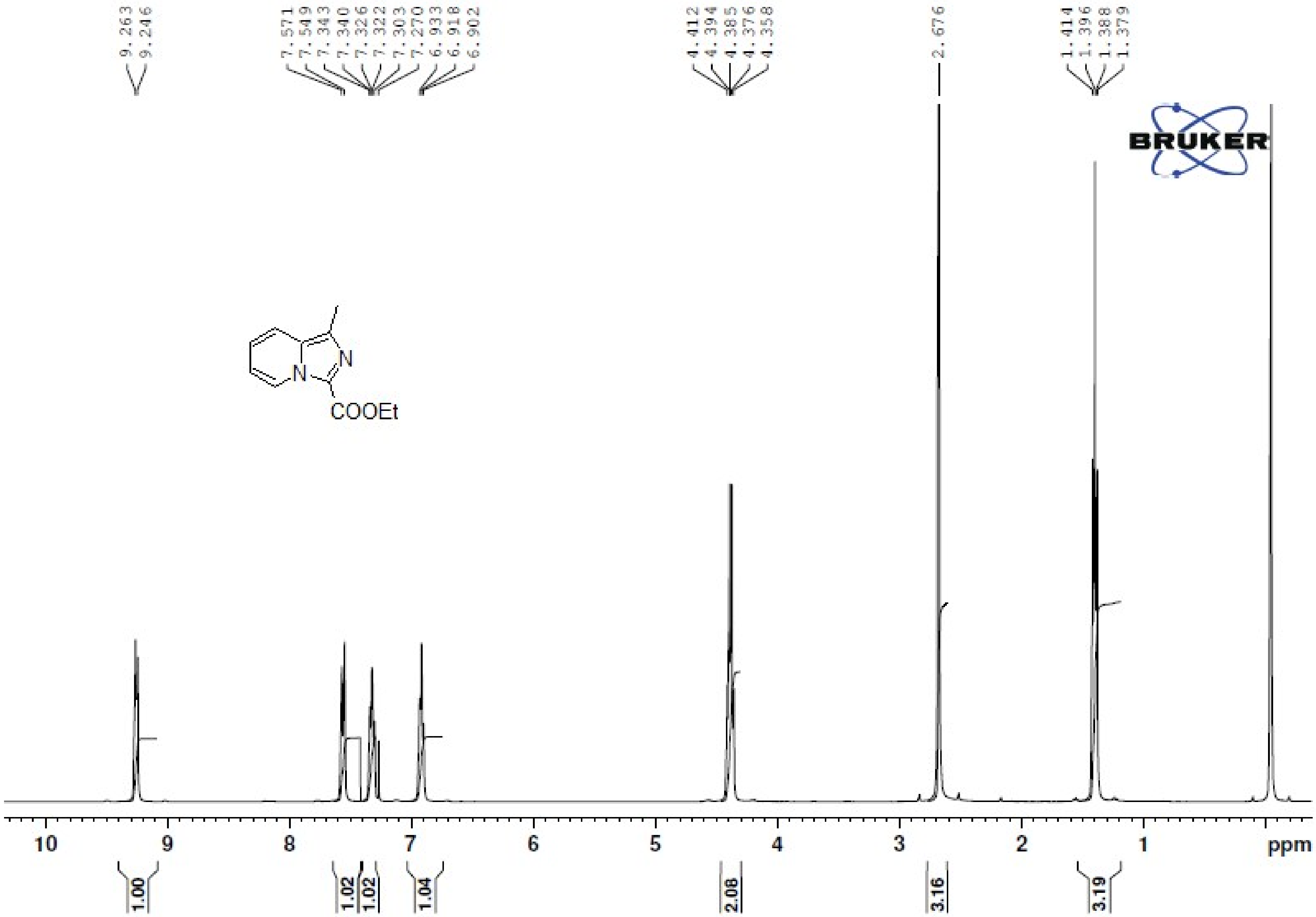

9) **^{1}H NMR spectra of Ethyl 1-methylimidazo[1,5-a]pyridine-3-carboxylate (3e)**

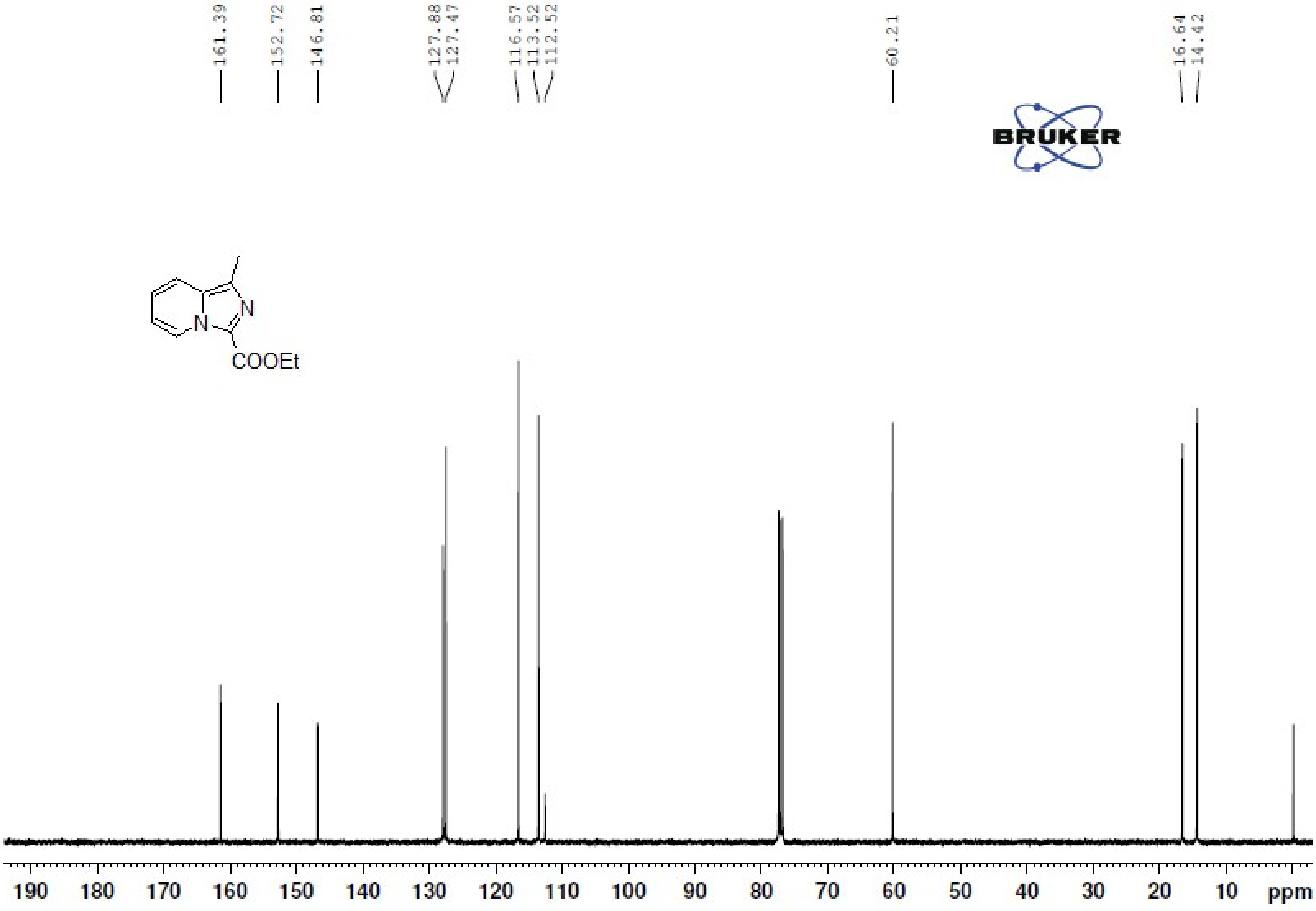

10) ^{13}C NMR spectra of Ethyl 1-methylimidazo[1,5-a]pyridine-3-carboxylate (3e)

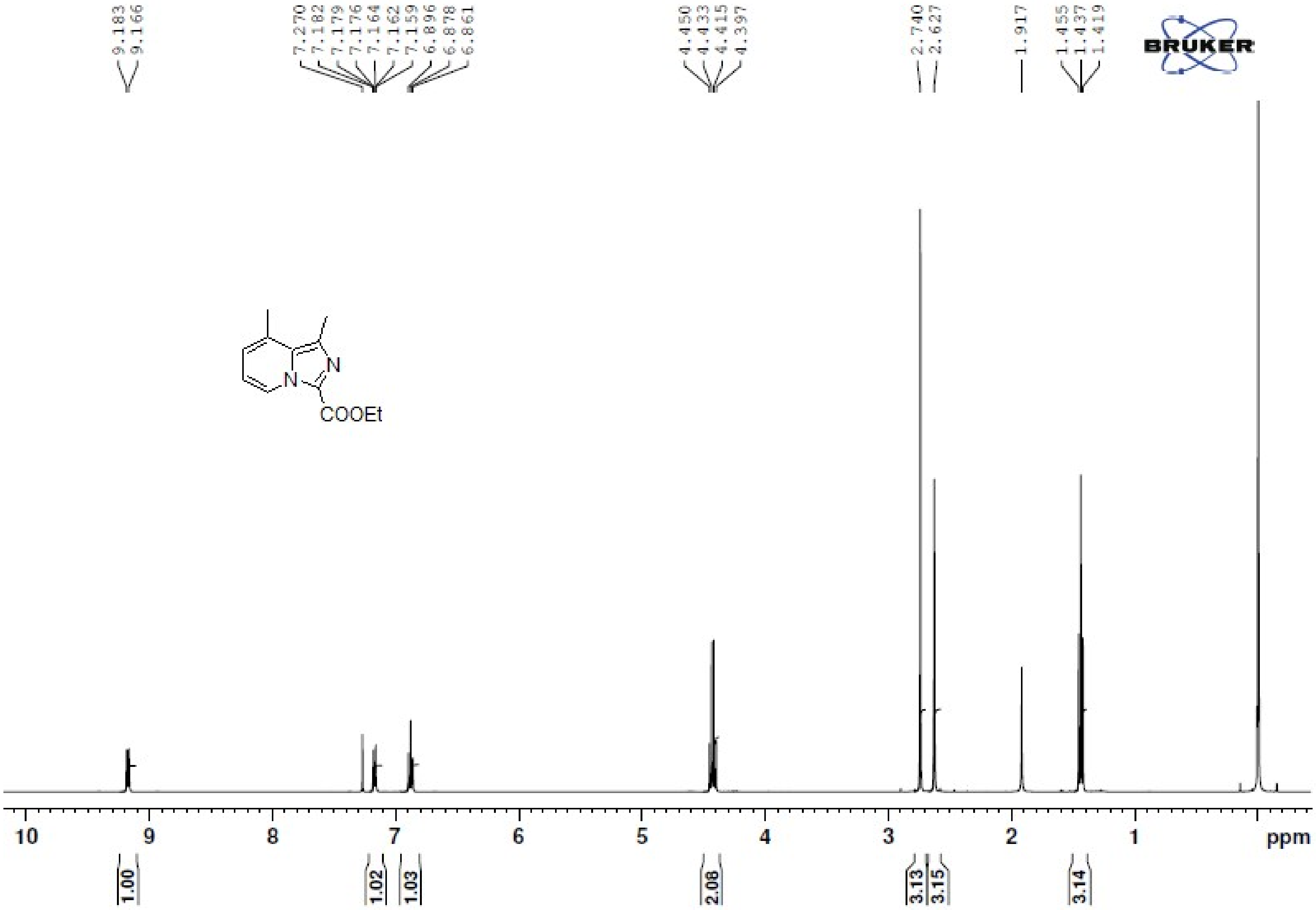

11) ^{1}H NMR spectra of Ethyl 1,8-dimethylimidazo[1,5-a]pyridine-3-carboxylate (3f)

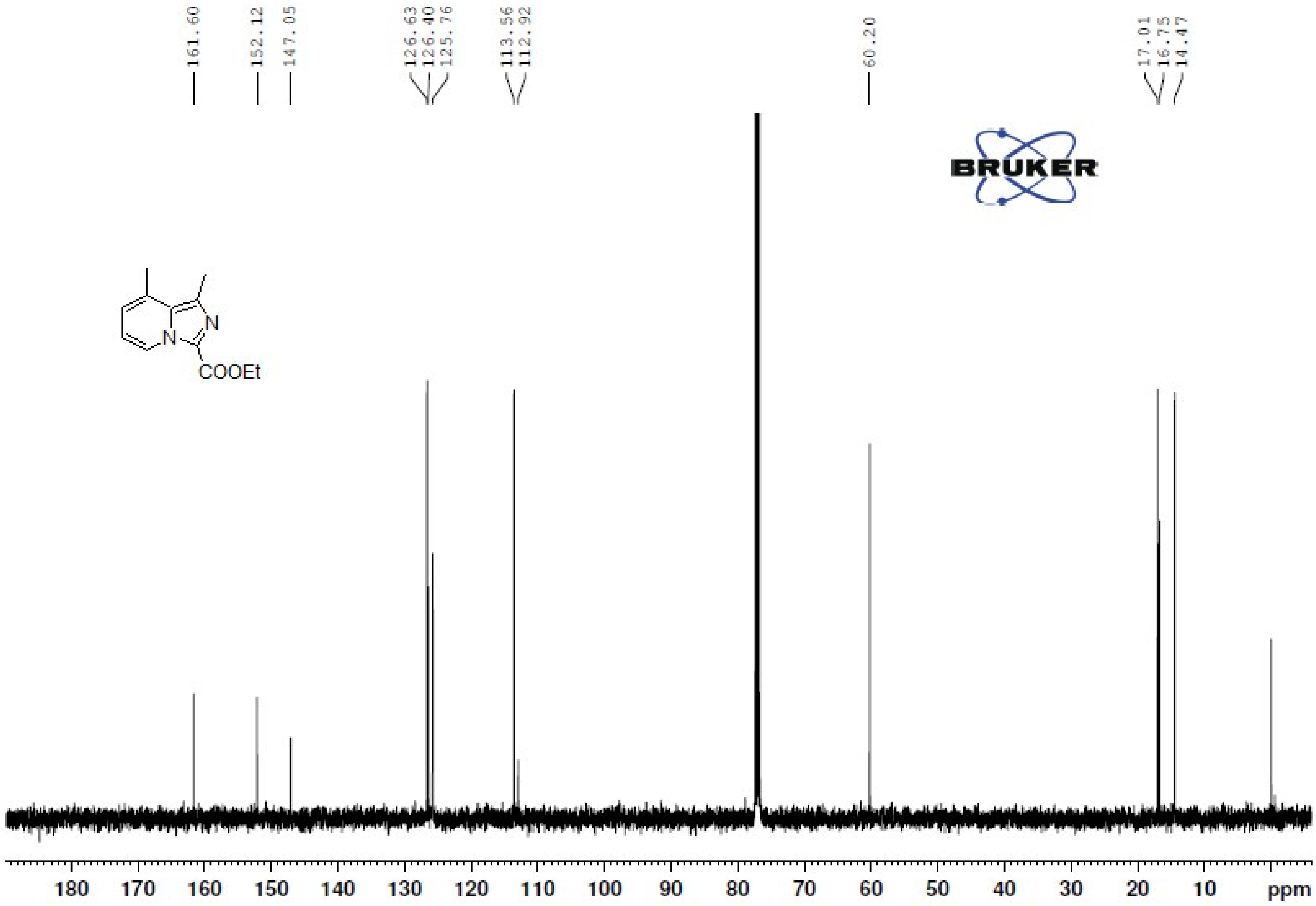

12) ^{13}C NMR spectra of Ethyl 1,8-dimethylimidazo[1,5-a]pyridine-3-carboxylate (3f)

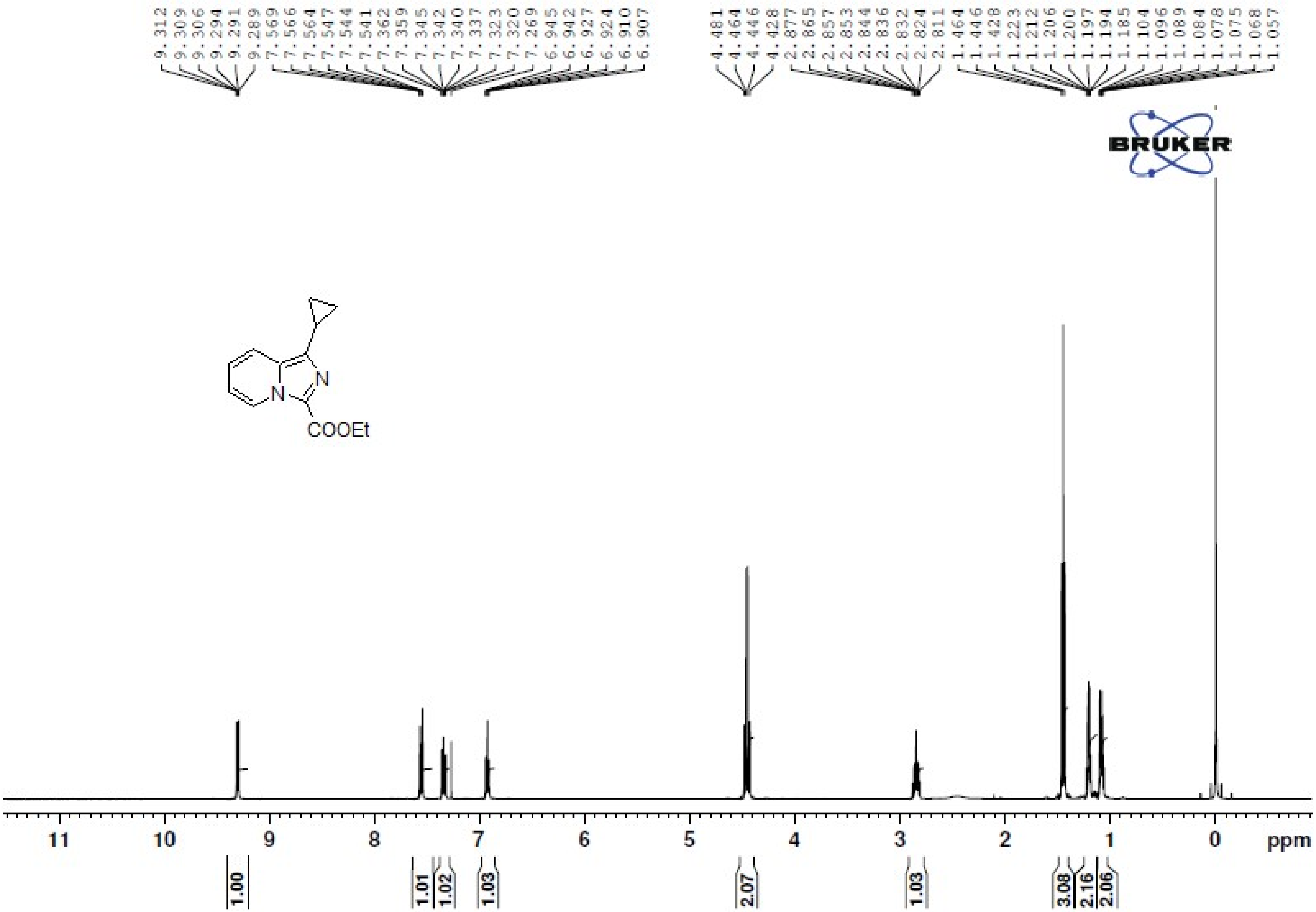

13) ^{1}H NMR spectra of Ethyl 1-cyclopropylimidazo[1,5-a]pyridine-3-carboxylate (3g)

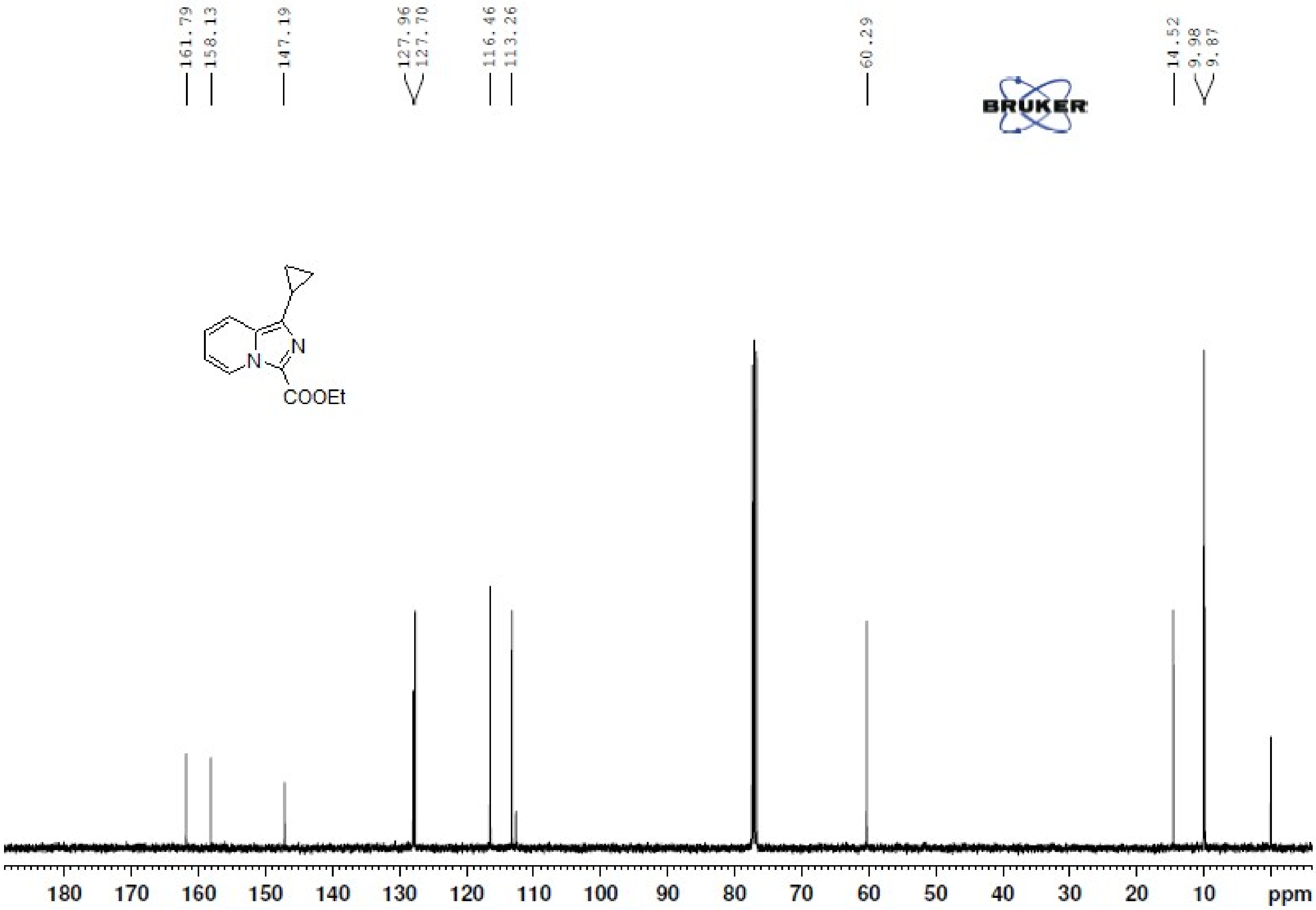

14) ^{13}C NMR spectra of Ethyl 1-cyclopropylimidazo[1,5-a]pyridine-3-carboxylate (3g)

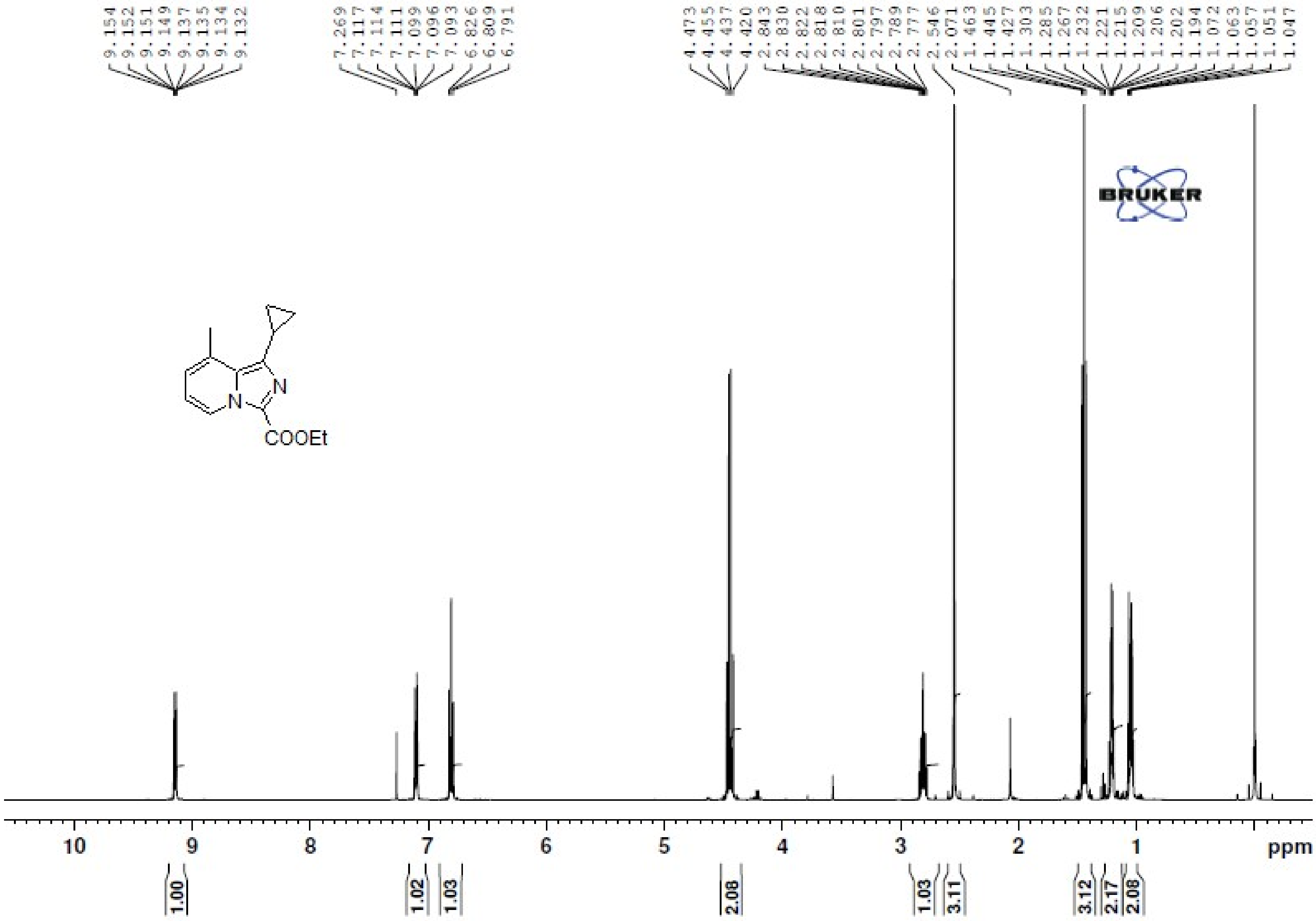

15) ^{1}H NMR spectra of Ethyl 1-cyclopropyl-8-methylimidazo[1,5-a]pyridine-3-carboxylate (3h)

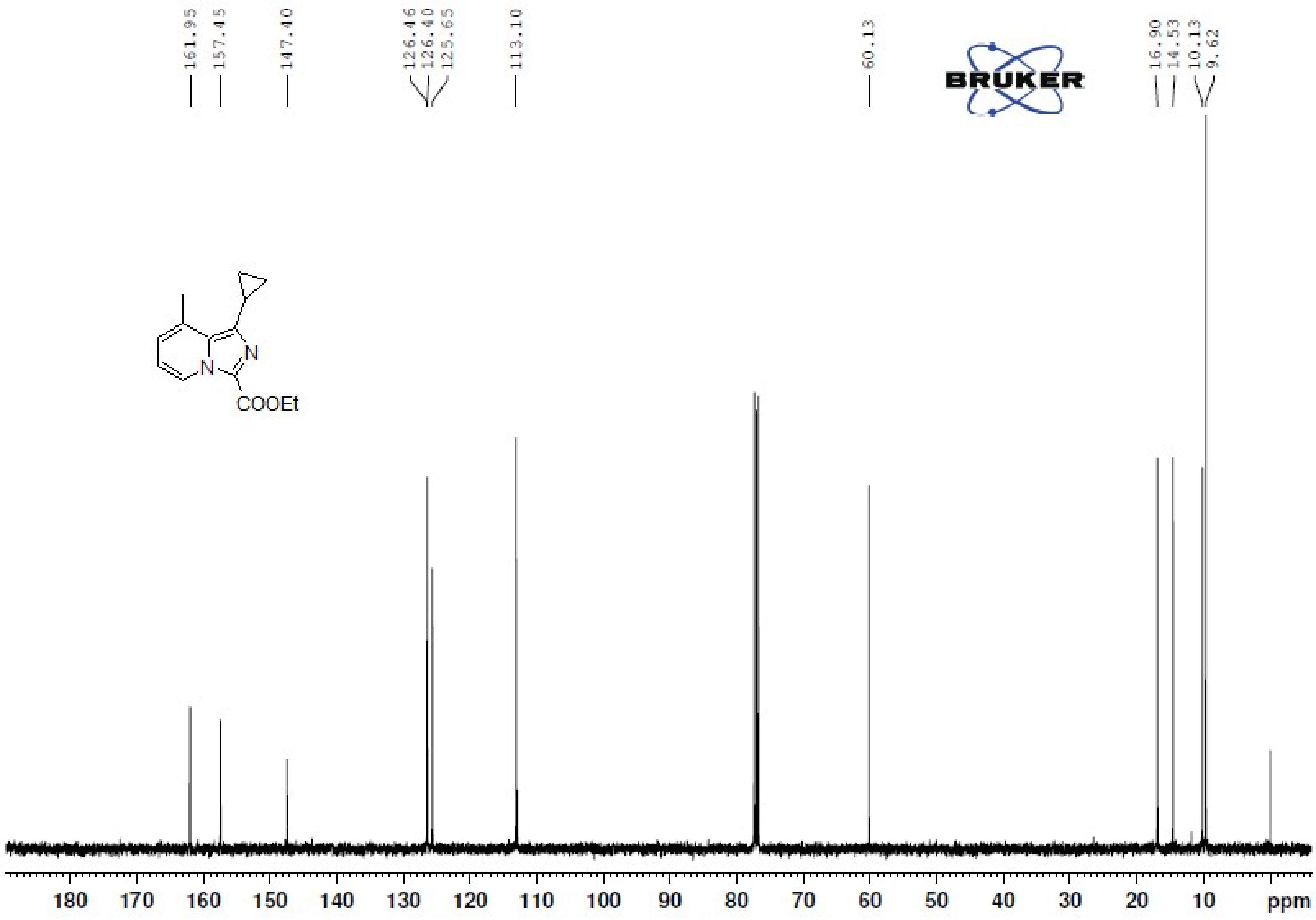

16) ^{13}C NMR spectra of Ethyl 1-cyclopropyl-8-methylimidazo[1,5-a]pyridine-3-carboxylate (3h)

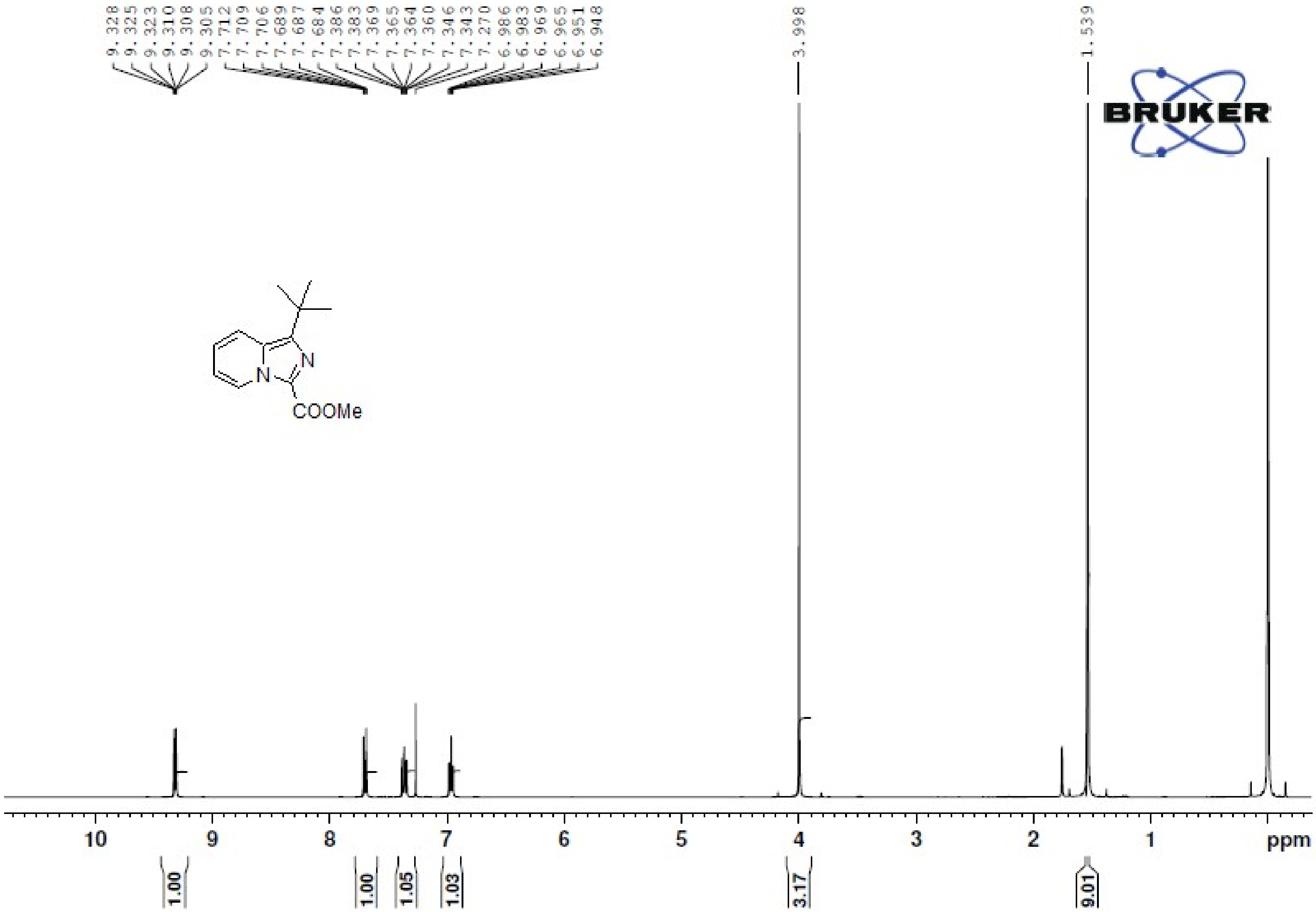

17) ^{1}H NMR spectra of Methyl 1-tert-butylimidazo[1,5-a]pyridine-3-carboxylate (3i)

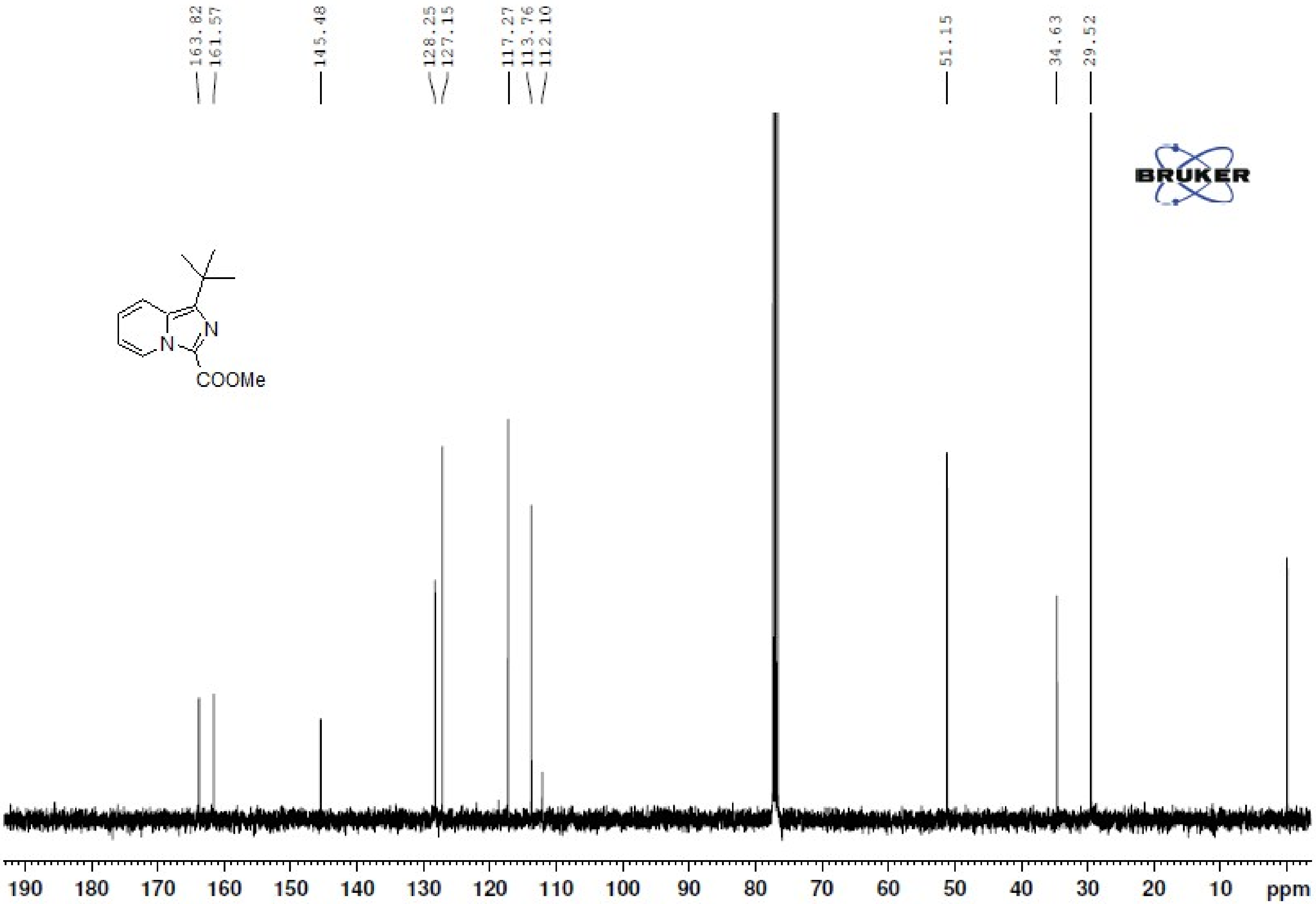

18) ^{13}C NMR spectra of Methyl 1-tert-butylimidazo[1,5-a]pyridine-3-carboxylate (3i)

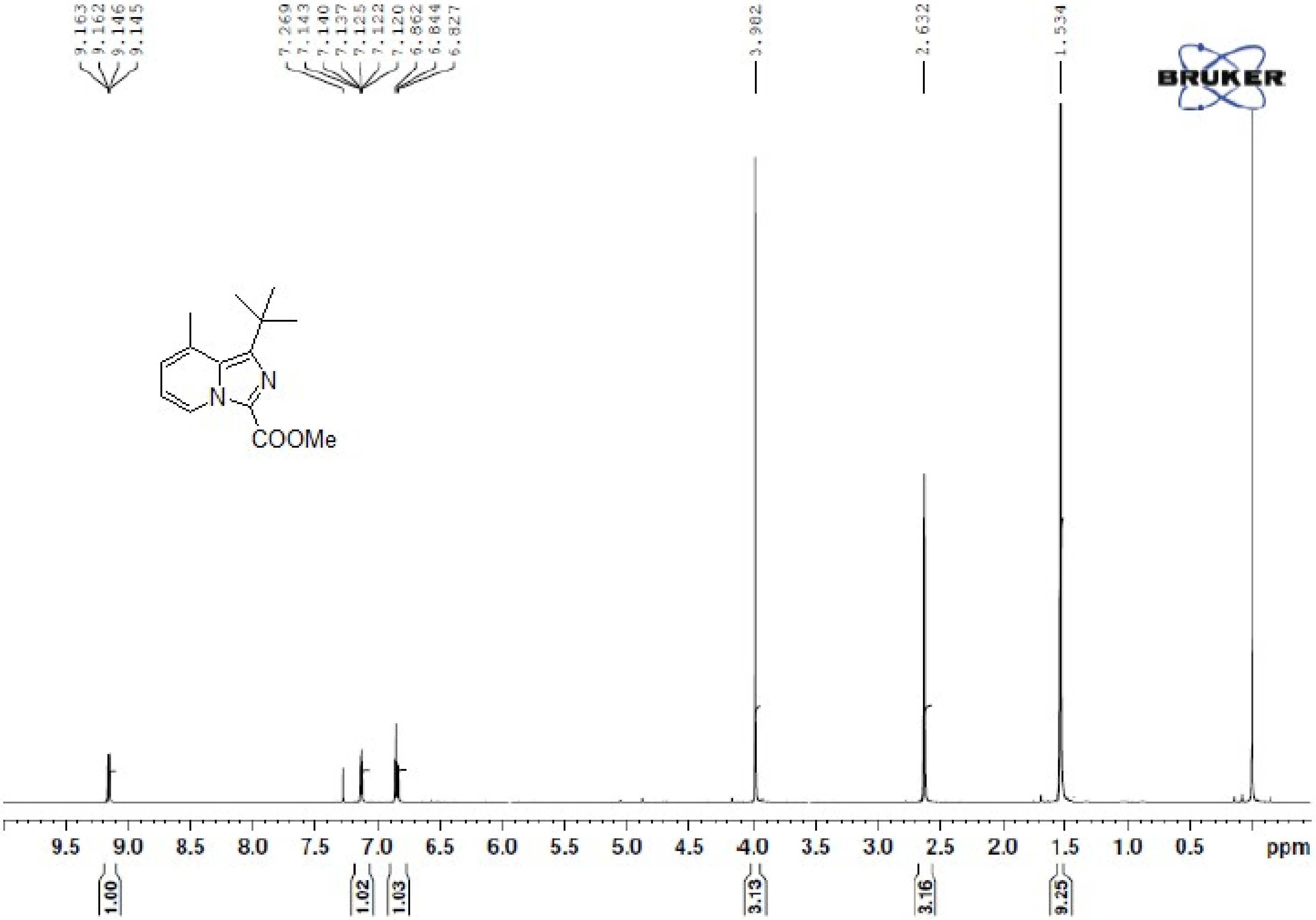

19) ¹H NMR spectra of Methyl 1-tert-butylimidazo[1,5-a]pyridine-3-carboxylate (3j)

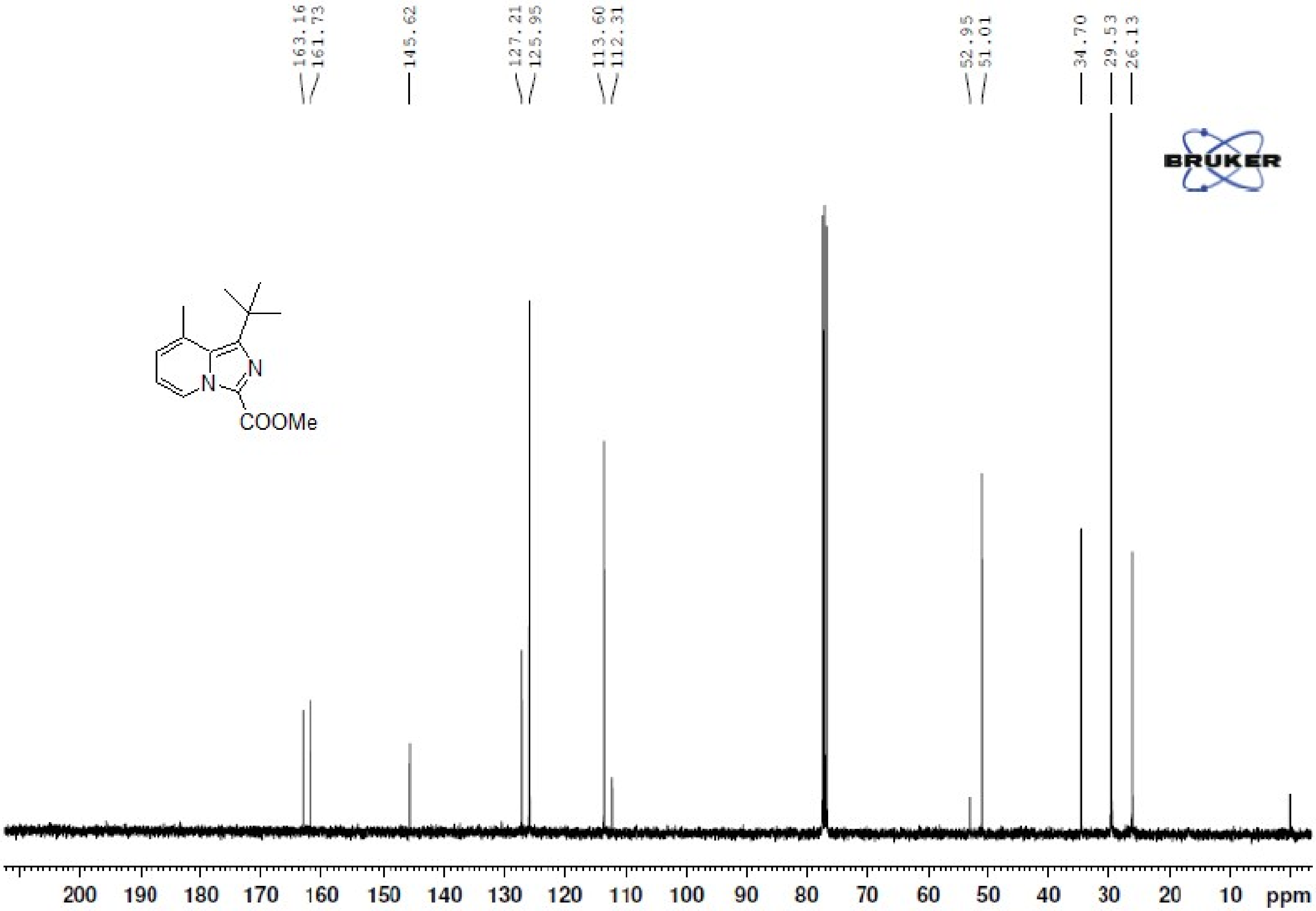

20) ^{13}C NMR spectra of Methyl 1-tert-butylimidazo[1,5-a]pyridine-3-carboxylate (3j)

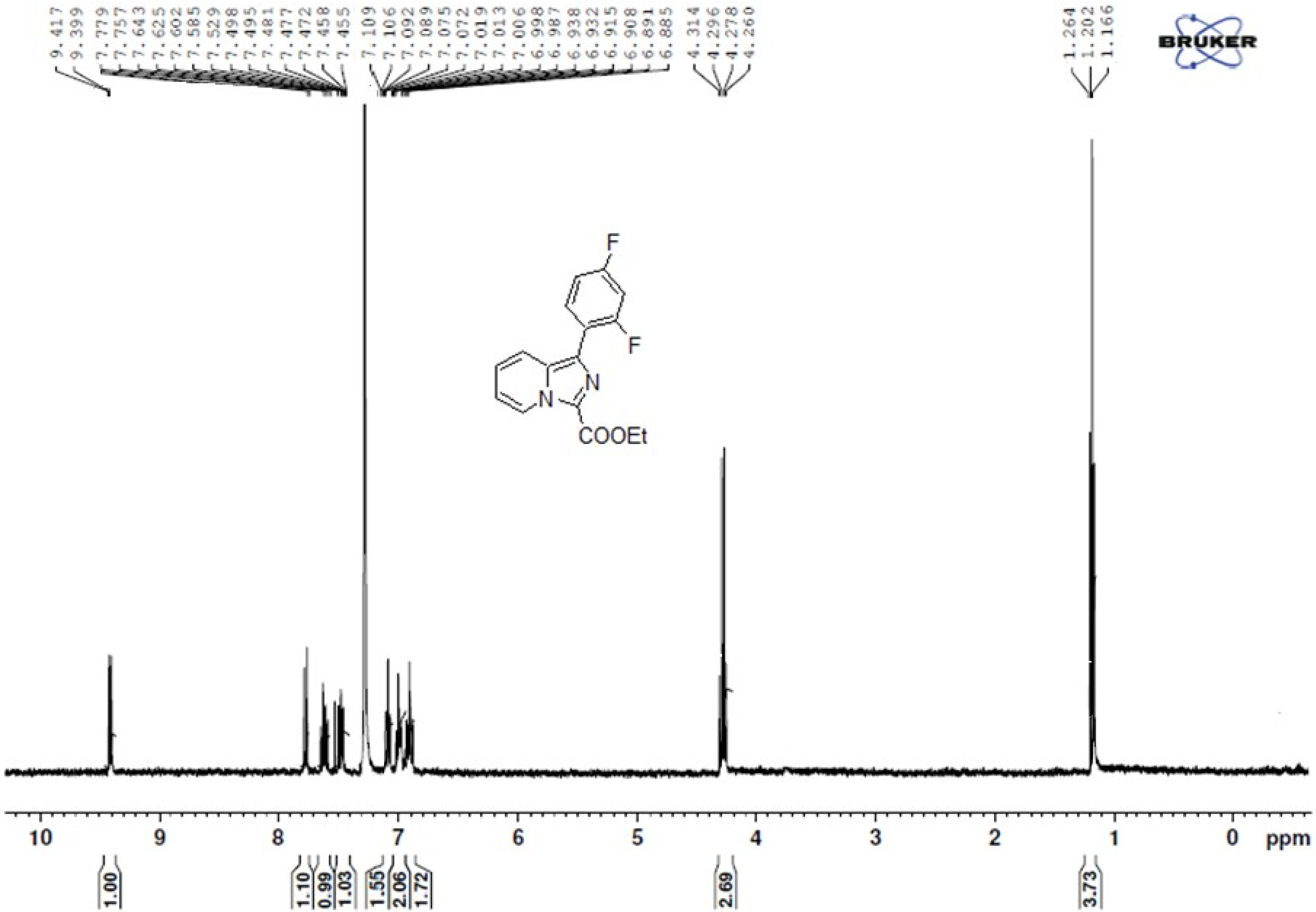

21) ^{1}H NMR spectra of Ethyl 1-(2,4-difluorophenyl)imidazo[1,5-a]pyridine-3-carboxylate (3k)

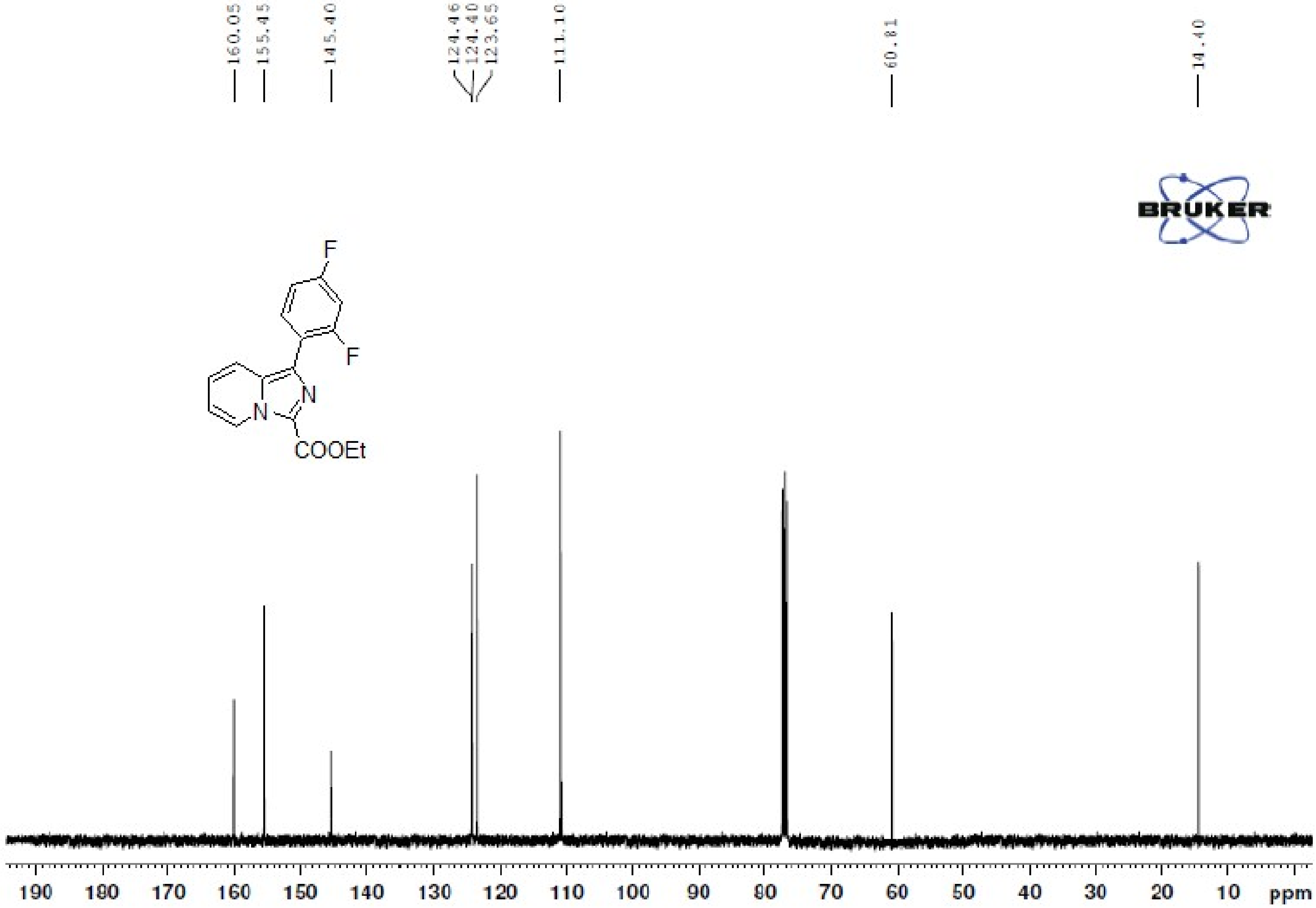

160.05
155.45
145.40
124.46
124.40
123.65
111.10
60.81
14.40
BRUKER
F
F
N
N
COOEt
190 180 170 160 150 140 130 120 110 100 90 80 70 60 50 40 30 20 10 ppm

22) ^{13}C NMR spectra of Ethyl 1-(2,4-difluorophenyl)imidazo[1,5-a]pyridine-3-carboxylate (3k)

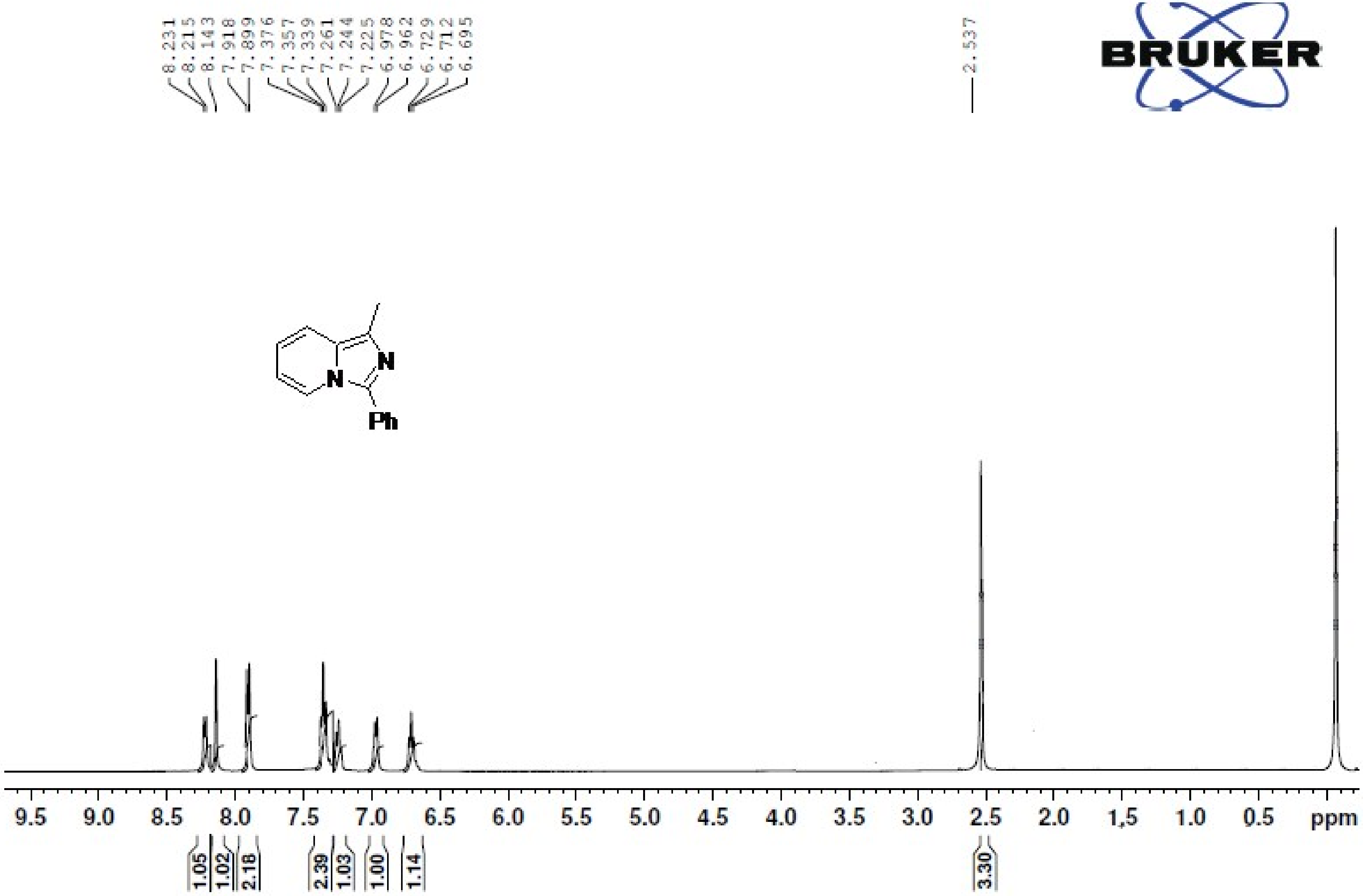

23) ^{1}H NMR spectra of 1-Methyl-3-phenylimidazo[1,5-a]pyridine (3m)

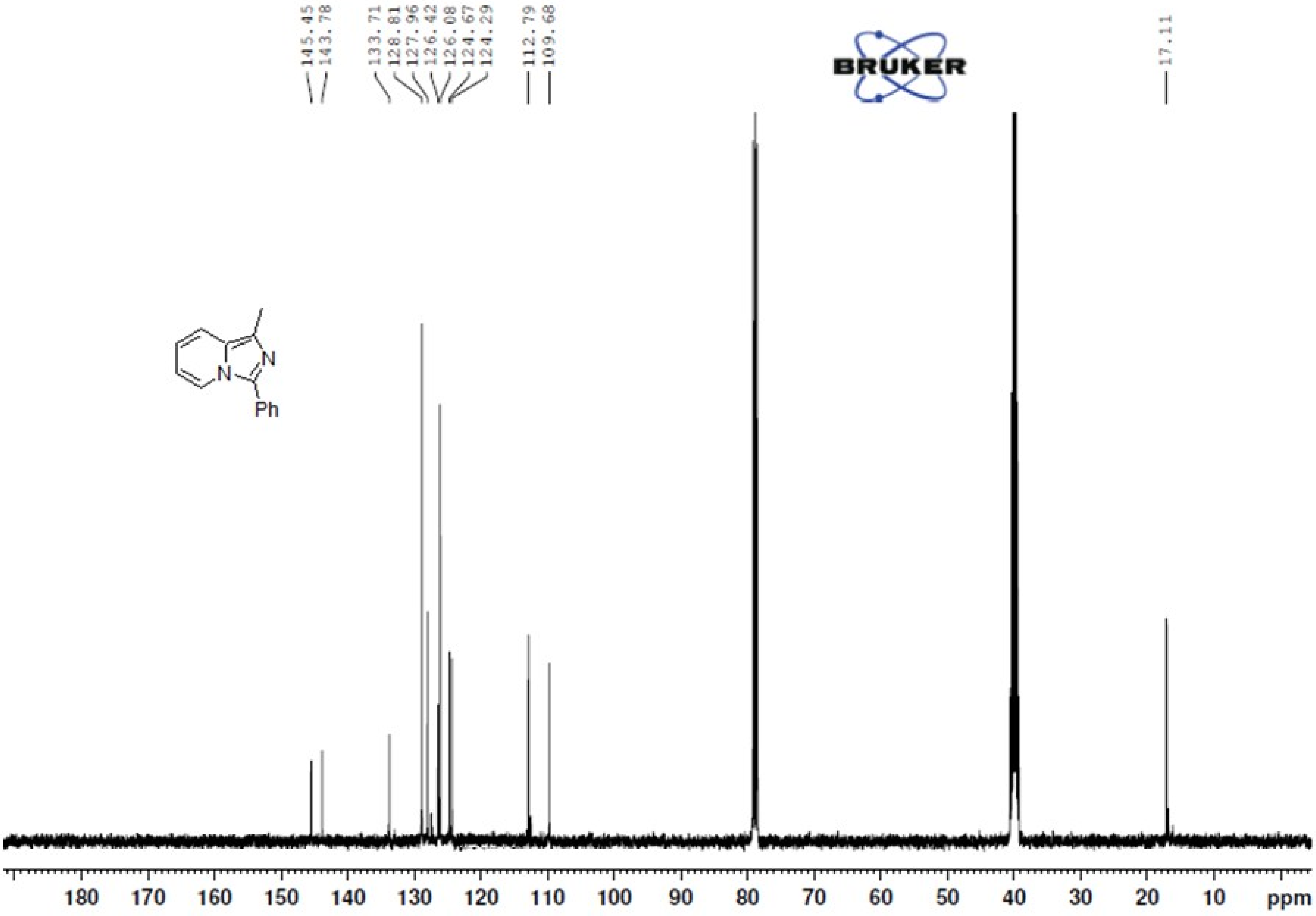

24) ^{13}C NMR spectra of 1-Methyl-3-phenylimidazo[1,5-a]pyridine (3m)
